# Dual Pharmacological Targeting of HDACs and PDE5 Inhibits Liver Disease Progression in a Mouse Model of Biliary Inflammation and Fibrosis

**DOI:** 10.3390/cancers12123748

**Published:** 2020-12-13

**Authors:** Alex Claveria-Cabello, Leticia Colyn, Iker Uriarte, Maria Ujue Latasa, Maria Arechederra, Jose M. Herranz, Laura Alvarez, Jesus M. Urman, Maria L. Martinez-Chantar, Jesus M. Banales, Bruno Sangro, Krista Rombouts, Julen Oyarzabal, Jose J. G. Marin, Carmen Berasain, Matias A. Avila, Maite G. Fernandez-Barrena

**Affiliations:** 1 Program of Hepatology, Center for Applied Medical Research (CIMA), University of Navarra, 31008 Pamplona, Spain; aclaveria.1@alumni.unav.es (A.C.-C.); lcolyn@alumni.unav.es (L.C.); iuriarte@unav.es (I.U.); mulatasa@unav.es (M.U.L.); macalderon@unav.es (M.A.); jmherranzalzueta@gmail.com (J.M.H.); lalvarez5@unav.es (L.A.); cberasain@unav.es (C.B.); 2 National Institute for the Study of Liver and Gastrointestinal Diseases (CIBERehd, Carlos III Health Institute), 28029 Madrid, Spain; mlmartinez@cicbiogune.es (M.L.M.-C.); jesus.banales@biodonostia.org (J.M.B.); bsangro@unav.es (B.S.); jjgmarin@usal.es (J.J.G.M.); 3 IdiSNA, Navarra Institute for Health Research, 31008 Pamplona, Spain; jm.urman.fernandez@navarra.es; 4 Department of Gastroenterology and Hepatology, Navarra University Hospital Complex, 31008 Pamplona, Spain; 5 Liver Disease Laboratory, Center for Cooperative Research in Biosciences (CIC bioGUNE), Basque Research and Technology Alliance (BRTA), Bizkaia Technology Park, 48160 Derio, Spain; 6 Department of Liver and Gastrointestinal Diseases, Biodonostia Health Research Institute, Donostia University Hospital, 20014 San Sebastian, Spain; 7 IKERBASQUE, Basque Foundation for Science, 48013 Bilbao, Spain; 8 Hepatology Unit, Department of Internal Medicine, University of Navarra Clinic, 31008 Pamplona, Spain; 9 Institute for Liver and Digestive Health, University College London, London NW3 2PF, UK; k.rombouts@ucl.ac.uk; 10 Program of Molecular Therapeutics, Center for Applied Medical Research (CIMA), University of Navarra, 31008 Pamplona, Spain; julenoyarzabal@external.unav.es; 11 Experimental Hepatology and Drug Targeting (HEVEPHARM), University of Salamanca, Biomedical Research Institute of Salamanca (IBSAL), 37007 Salamanca, Spain

**Keywords:** liver fibrosis, hepatobiliary carcinogenesis, histone deacetylases, cGMP phosphodiesterase inhibitor, HDAC inhibitor, precision medicine

## Abstract

**Simple Summary:**

Chronic liver injury and inflammation leads to excessive deposition of extracellular matrix, known as liver fibrosis, and the distortion of the hepatic parenchyma. Liver fibrosis may progress to cirrhosis, a condition in which hepatic function is impaired and most cases of liver tumors occur. Currently, there are no effective therapies to inhibit and reverse the progression of liver fibrosis, and therefore, chronic liver disease remains a global health problem. In this study we have tested the efficacy of a new class of molecules that simultaneously target two molecular pathways known to be involved in the pathogenesis of hepatic fibrosis. In a clinically relevant mouse model of liver injury and inflammation we show that the combined inhibition of histones deacetylases and the cyclic guanosine monophosphate (cGMP) phosphodiesterase phosphodiesterase 5 (PDE5) results in potent anti-inflammatory and anti-fibrotic effects. Our findings open new avenues for the treatment of liver fibrosis and therefore, the prevention of hepatic carcinogenesis.

**Abstract:**

Liver fibrosis, a common hallmark of chronic liver disease (CLD), is characterized by the accumulation of extracellular matrix secreted by activated hepatic fibroblasts and stellate cells (HSC). Fibrogenesis involves multiple cellular and molecular processes and is intimately linked to chronic hepatic inflammation. Importantly, it has been shown to promote the loss of liver function and liver carcinogenesis. No effective therapies for liver fibrosis are currently available. We examined the anti-fibrogenic potential of a new drug (CM414) that simultaneously inhibits histone deacetylases (HDACs), more precisely HDAC1, 2, and 3 (Class I) and HDAC6 (Class II) and stimulates the cyclic guanosine monophosphate (cGMP)-protein kinase G (PKG) pathway activity through phosphodiesterase 5 (PDE5) inhibition, two mechanisms independently involved in liver fibrosis. To this end, we treated *Mdr2*-KO mice, a clinically relevant model of liver inflammation and fibrosis, with our dual HDAC/PDE5 inhibitor CM414. We observed a decrease in the expression of fibrogenic markers and collagen deposition, together with a marked reduction in inflammation. No signs of hepatic or systemic toxicity were recorded. Mechanistic studies in cultured human HSC and cholangiocytes (LX2 and H69 cell lines, respectively) demonstrated that CM414 inhibited pro-fibrogenic and inflammatory responses, including those triggered by transforming growth factor β (TGFβ). Our study supports the notion that simultaneous targeting of pro-inflammatory and fibrogenic mechanisms controlled by HDACs and PDE5 with a single molecule, such as CM414, can be a new disease-modifying strategy.

## 1. Introduction

Liver fibrosis, characterized by excessive accumulation of extracellular matrix (ECM) components, is the principal event that contributes to most of the complications of chronic liver diseases (CLDs), and represents an important and underestimated global health problem [1,2]. The progression of CLD is usually a longstanding process, as many liver pathologies develop, on average, after 15–20 years of chronic parenchymal injury [3]. Epidemiological studies have estimated that more than 800 million individuals worldwide are affected by a form of CLD, with a mortality rate of two million deaths per year [4,5,6,7]. The most relevant etiologies leading to CLD comprise chronic infection by hepatitis B and C viruses (HBV and HCV), non-alcoholic fatty liver disease (NAFLD), excess ethanol consumption, autoimmune diseases, including conditions affecting the biliary tree such as primary biliary cholangitis (PBC), and primary sclerosing cholangitis (PSC), autoimmune hepatitis (AIH), and rare hereditary diseases such as Wilson’s disease, α1-anti-trypsin deficiency, or hemochromatosis [4,5,8].

The main event from the beginning of CLD is hepatocellular death that triggers an inflammatory reaction. This process is linked to a potent regenerative response in order to restore the lost hepatic tissue. In the context of acute or self-limited damage, this inflammatory and wound-healing response is transient, and the liver architecture is restored to the normal stage. However, under chronic injury, this response is sustained, leading to the accumulation of ECM and the progressive substitution of liver parenchyma by scar tissue. Persistent inflammation and fibrosis, together with the presence of regenerative nodules of hepatocytes, are hallmarks of liver cirrhosis. The cirrhotic state is also characterized by hepatocellular dedifferentiation and the loss of parenchymal function [9]. In this context is where most cases of primary liver cancer, mainly hepatocellular carcinoma (HCC) but also cholangiocarcinoma (CCA), develop and therefore, liver cirrhosis is considered a pre-neoplastic condition [8,10,11].

Activated myofibroblasts are considered the critical effectors of fibrosis development, representing a spectrum of ECM-producing cells that mainly derive from hepatic stellate cells (HSC) and portal fibroblasts [12,13]. Following liver injury, these cells acquire a phenotype characterized by the loss of retinoid droplets and contractile activity, increased proliferation, production of ECM, and the release of pro-inflammatory, pro-fibrogenic, and pro-mitogenic cytokines. Myofibroblast trans-differentiation is, therefore, a highly conserved and tightly regulated process where coordinated cellular changes result from either activating events as well as from the loss of repressive signaling [14,15]. A chronic inflammatory response is critically sustained during CLD progression [16]. Liver cells death and inflammation initiates fibrogenesis activating myofibroblasts and resident liver macrophages (Kupffer cells), and by recruiting monocytes and other immune cells from peripheral blood. The release of several soluble peptide mediators (cytokines, growth factors, and chemokines) and reactive oxygen species (ROS) generation influence the trafficking of inflammatory and immune cells within the liver. Therefore fibrosis progression is the net result of many diverse and regulated actions [17].

Despite significant advances in the understanding of the cellular and molecular mechanisms of liver fibrogenesis, no effective pharmacological therapies have reached the clinical practice yet [2]. To date, the most effective therapy is to remove the causative agent (e.g., eliminating viral infection or stopping alcohol consumption), or, in the case of advanced stages, liver transplantation [18,19]. This situation highlights the urgent need for new approaches to identify effective therapeutic strategies.

We and others have previously demonstrated that epigenetic events are fundamental in myofibroblast activation and functions [20,21,22]. The term epigenetics describes reversible mechanisms controlling gene expression that can be inherited through cell division and that do not involve alterations to the underlying DNA sequence. Epigenetics includes at least three mechanisms: DNA methylation, histone modifications, and non-coding RNA (ncRNA) mediated gene regulation [23]. The modulation of gene expression is not only important for myofibroblasts activation, but also for their ability to respond to microenvironmental cues and their persistence in CLD up to cancer development [21,24]. Histone acetylation is one of the most studied epigenetic events, a reversible process closely linked to gene transcriptional activation, while histone deacetylation consistently results in gene transcriptional repression [25]. The balance between the acetylated and deacetylated states of histones is controlled by the antagonistic actions of two types of enzymes: Histone acetyltransferases (HATs) and histone deacetylases (HDACs) [26]. HDACs have emerged as crucial transcriptional regulators in diverse physiological aspects of liver fibrosis [27]. Members of the HDAC superfamily have been classified according to their sequence features and domain organization. Class I HDACs, including HDAC1, 2, 3, and 8, are ubiquitously expressed and reside in the nucleus while Class II HDACs (HDAC4, 5, 6, 7, 9, and 10) are cell-type specific and able to shuttle between cytoplasm and nucleus. Class III HDACs, which are structurally and enzymatically distinct, are named sirtuins (SIRT 1-7), present different localizations including the nucleus, cytoplasm, or mitochondria, and finally, Class IV HDACs, composed only by HDAC11, localizes to the nucleus and shares sequence homology with the catalytic domains of Class I and II HDACs [28]. It has been demonstrated that the inhibition of Class I and II HDACs leads to a noticeable reduction of HSC activation and proliferation [29], and the induction of apoptosis and autophagic cell death of activated HSCs [30]. These HDACs also participate in the regulation of transforming growth factor β (TGFβ) signaling, a key fibrogenic pathway, and of diverse inflammatory responses [27]. Moreover, it has been well established how specific HDACs catalyze the deacetylation of many non-histone proteins, thus being able to affect other different cellular processes besides gene transcription [31], as is the case of HDAC6. HDAC6 localizes predominantly to the cytoplasm, it is structurally and functionally unique, distinguishing itself from other family members in that it contains two HDAC domains and a ubiquitin-binding motif, the BUZ finger [32]. HDAC6 regulates microtubule function and stability via α-tubulin deacetylation [33,34], one of its best-characterized substrates, and controls cell growth [35], inflammatory events [36,37], and fibrogenic mechanisms [38,39,40]. HDAC inhibitors (HDACi) have become promising drug candidates, presenting pleiotropic anti-fibrogenic properties depending on their potency and specific activity against the different classes of HDACs [27,41]. Several non-selective HDACi have been approved by the Food and Drug Administration (FDA) as antitumor agents [42,43]. However, many of them harbor undesired side effects, including a marked hepatotoxicity [44], so none of them are currently under investigation for liver fibrosis. Therefore, targeting specific HDACs with higher selectivity and a better understanding of the role of each isozyme could lead the generation more effective and less toxic therapeutic agents for this condition.

Another interesting group of compounds that has recently shown promising anti-fibrogenic properties is that of phosphodiesterase 5 inhibitors (PDE5i) [45,46]. PDE5i are already approved for the treatment of erectile dysfunction [47], lower urinary tract disorders, and pulmonary arterial hypertension [48]. Phosphodiesterase 5 (PDE5) acts by hydrolyzing the phosphodiester bond of the second messenger cyclic guanosine monophosphate (cGMP), converting it to the inactive GMP. PDE5 inhibition thus results in increased intracellular levels of cGMP and the activation of downstream signaling cascades, including protein kinase G (PKG)-mediated signaling. Synthesis of cGMP is catalyzed by the intracellular soluble guanylate cyclase (sGC), which can be activated by NO binding [49]. It has been observed that in liver fibrosis exists a profound perturbation of the NO-sGC-cGMP signaling axis. This is due in part to a defective availability and responsiveness to NO by activated HSC cells. NO can be depleted by increased ROS levels or the impaired expression of enzymes implicated in its synthesis, such as endothelial NO synthase (NOS) [50,51], while its cellular effects may be dampened by reduced expression of PKG [52]. In this sense, strategies directed to increase intracellular cGMP levels through the use of PDE5i as well as sGC stimulators are demonstrating potent anti-inflammatory and anti-fibrogenic properties [53,54,55].

**Figure 1:**
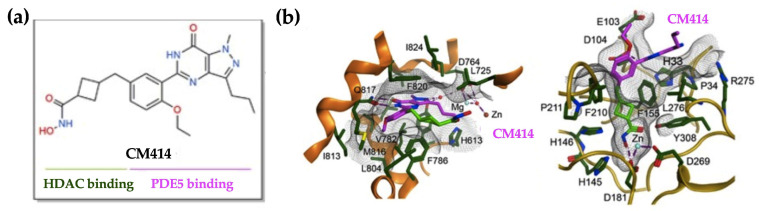
CM414, dual inhibitor of Class I histone deacetylases (HDACs) and phosphodiesterase 5 (PDE5). (**a**) CM414 chemical formula highlighting the hydroxamic moiety that interacts with HDACs and the pyrazolopyrimidinone core that interacts with PDE5. (**b**) representations of CM414 bound to PDE5 (left panel) and HDAC2 (right panel).

Based on all these observations, we hypothesized that simultaneous interference with the enzymatic activities of HDACs and PDE5 could be a potential strategy to halt fibrosis progression. Therefore, we tested the effects of CM414, a recently developed first-in-class small-molecule with moderate inhibitory activity against Class I HDACs but a potent inhibitor of HDAC6 and PDE5 (Figure 1). CM414 was originally conceived to be applied in the context of neurological pathologies such as Alzheimer disease [56]. Previous studies revealed that the co-administration of sub-effective doses of a PDE5i together with vorinostat, an HDACi, produced a synergistic effect on the induction of epigenetic responses (i.e., increased H3K9Ac levels) and prevented neurodegenerative events [57]. These findings suggested that pathways affected by these two inhibitors could functionally interact at some point in the regulation of gene expression. CM414 has an acceptable therapeutic window in vitro and in vivo, with no evidences of toxicity observed [56,58].

To investigate the potential anti-fibrogenic effects of CM414, we used a clinically relevant animal model of chronic portal inflammation and bile duct proliferation with progression to liver fibrosis and cancer (*Mdr2/Abcb4*-deficient mice (*Mdr2*-KO)). *Mdr2*-KO mice lack the ABC protein Mdr2, a phosphatidylcholine transporter, which results in impaired phospholipid biliary secretion. The absence of phospholipids able to neutralize the detergent activity of bile acids leads to an unspecific attack to the canalicular and ductular membranes resulting in bile regurgitation into the portal tracts [59], cell death, and portal inflammation [60,61]. All these effects appear in *Mdr2*-KO mice already within the first week of age, accompanied by an increase in serum transaminases levels and followed by enhanced ECM deposition and progression to fibrosis [62]. As a consequence of chronic inflammation and fibrosis, *Mdr2*-KO mice have been shown to develop HCC within 12–15 months of age [63].

Using this model of CLD, here we show that CM414 administration was able to attenuate liver injury and inflammation and to inhibit the progression of CLD in *Mdr2*-KO mice with established fibrosis without apparent toxicity. Mechanistic studies identified different and cooperative actions of CM414 resulting from the simultaneous inhibition of Class I HDACs, HDAC6, and PDE5.

Together, our findings indicate that CM414 may represent an excellent example of developing new anti-fibrogenic and cancer preventive strategies based on multitargeted drugs with anti-inflammatory and anti-fibrogenic properties. This work may pave the way for future treatments of fibrosis, a condition that not only affects the liver as it also occurs in other organs like the lung and kidney, with dramatic consequences.

## 2. Results

### 2.1. In Vivo Evaluation of the Anti-Fibrogenic Potential of CM414, a Dual HDAC-PDE5 Inhibitor

**Figure 2:**
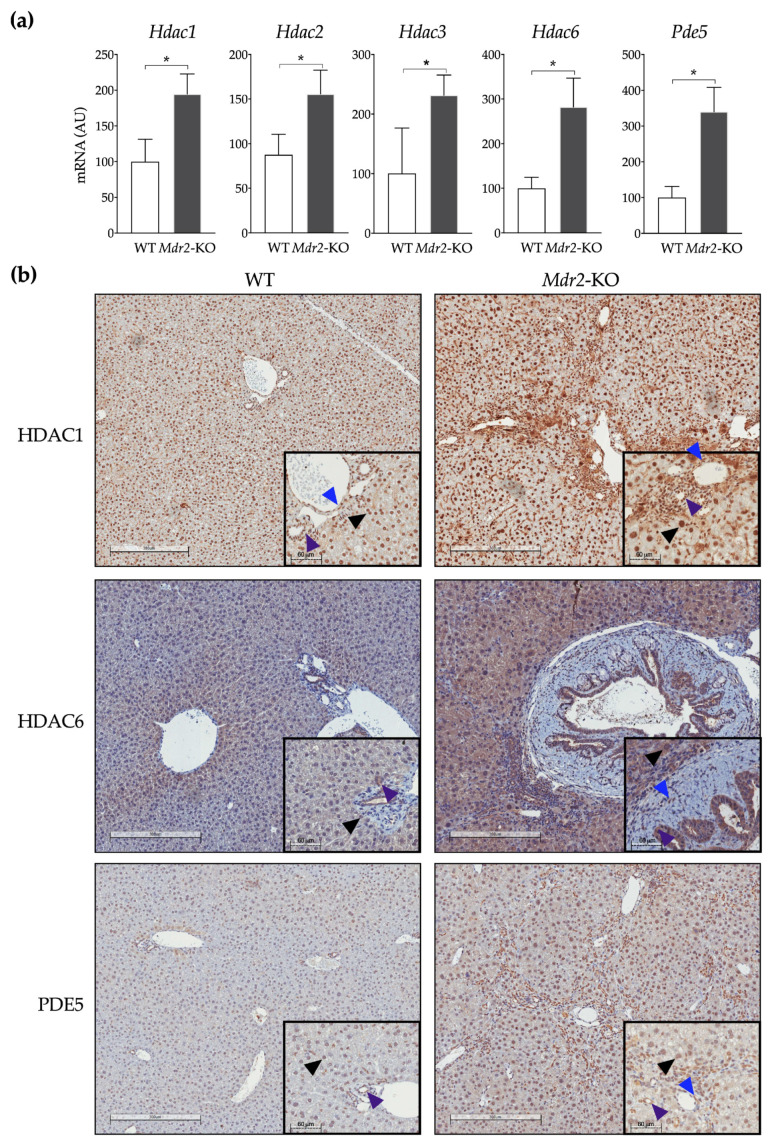
Expression of CM414 molecular targets in the livers of six-months old WT and *Mdr2*-KO mice. (**a**) qPCR analyses of *Hdac1*, *Hdac2*, *Hdac3*, *Hdac6,* and *Pde5* mRNA levels. (**b**) representative images of HDAC1, HDAC6, and PDE5 immunostaining in livers from WT and *Mdr2*-KO mice. Insets show enlarged areas with arrowheads indicating positive staining in hepatocytes (black), fibrogenic cells (blue), or biliary cells (purple). * *p* < 0.05.

*Mdr2*-KO mice are a widely accepted and clinically relevant genetic mouse model of liver fibrosis and carcinogenesis [61,63]. By two months of age, these mice already display a significant degree of periportal fibrosis in an environment of ongoing liver injury and inflammation [64]. To test our dual HDAC and PDE5 inhibitor, we chose to use older mice with a more advanced stage of the disease to better mimic a therapeutic approach rather than a preventive strategy. First, we examined the expression of CM414 molecular targets in the livers from six-month-old *Mdr2*-WT and *Mdr2*-KO mice. *Mdr2*-KO mice showed significantly elevated expression levels of the Class I *Hdac* enzymes *Hdac1*, *Hdac2*, and *Hdac3* in liver tissues as analyzed by qPCR. The expression of *Hdac6* and *Pde5* was also evaluated, and their mRNA levels were also significantly increased compared to those in healthy controls (Figure 2a). Next, we performed immunohistochemical staining of liver tissue samples from these mice. We detected the presence of HDAC1 in the nucleus of most hepatic cells, including activated myofibroblasts and cholangiocytes in *Mdr2*-KO mice (Figure 2b). HDAC6 showed cytoplasmic localization, whose abundance was markedly increased in cholangiocytes in *Mdr2*-KO mice, but also in hepatocytes and myofibroblasts. In healthy livers, PDE5 protein was mainly expressed by perivenular hepatocytes and, to a lesser extent, by perisinusoidal cells. In contrast, in *Mdr2*-KO livers, hepatic staining of PDE5 increased significantly, particularly in cholangiocytes and fibrous septa (Figure 2b).

**Figure 3:**
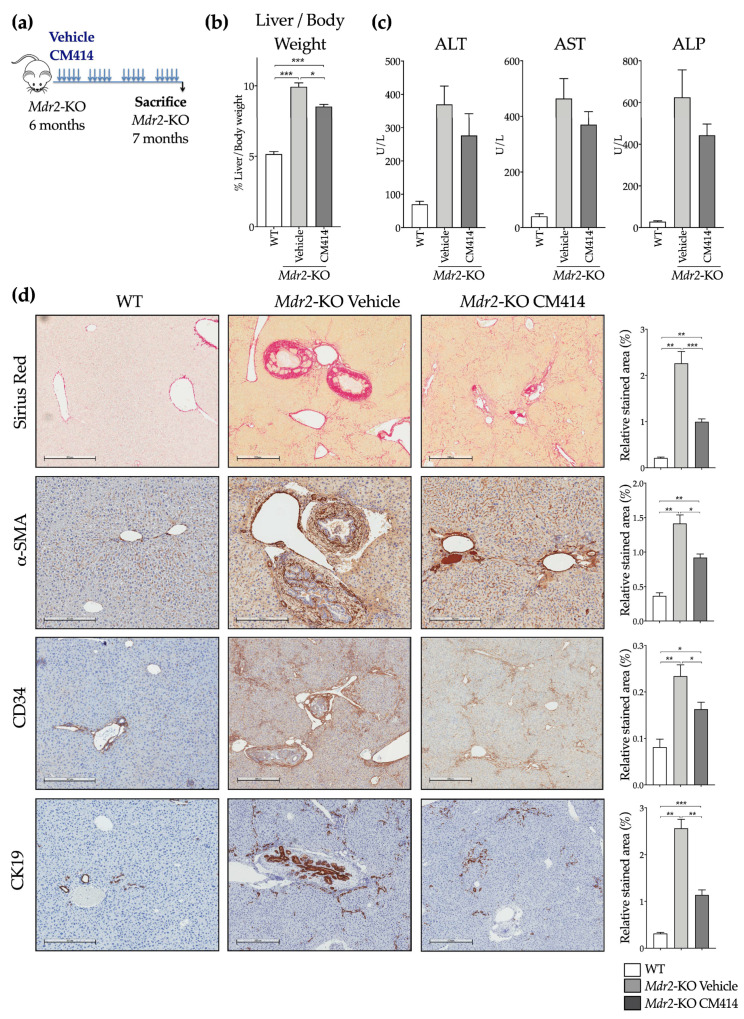
Effect of CM414 treatment on liver injury and fibrosis in *Mdr2*-KO mice. (**a**) aiagram showing the treatment schedule of *Mdr2*-KO mice. Animals received a daily i.p. injection of vehicle or CM414 (40 mg/kg body weight) five days per week for 4 weeks, when animals were sacrificed, and serum and liver tissue samples were analyzed. (**b**) liver-to-body weight ratio (expressed as %) in WT mice and in *Mdr2*-KO mice treated with vehicle or CM414. (**c**) serum transaminases (alanine aminotransferase (ALT), aspartate aminotransferase (AST)) and alkaline phosphatase (ALP) levels in WT and *Mdr2*-KO mice treated with vehicle or with CM414. (**d**) representative images of Sirius Red staining for collagen and immunostaining for α-SMA, CD34, and CK19 in liver sections from WT and *Mdr2*-KO mice treated with vehicle or with CM414. Graphs on the right show the corresponding quantification of positively stained areas. * *p* < 0.05. ** *p* < 0.01. *** *p* < 0.001.

To evaluate the effects of CM414, six-month-old *Mdr2*-KO mice were treated with 40 mg/kg (i.p.) of the drug for 1 month as indicated (Figure 3a). CM414 administration was well tolerated, and treated animals gained similar weight as those administered with the corresponding vehicle (data not shown). We found a reduced liver to body weight ratio (Figure 3b) and improved aspartate aminotransferase (AST), alanine aminotransferase (ALT), and alkaline phosphatase (ALP) serum levels in the CM414 treated group (Figure 3c). As expected, six months-old-*Mdr2*-KO mice already presented advanced fibrosis as indicated by Sirius Red staining of crosslinked collagen in liver sections (Figure 3d). CM414 treatment markedly reduced collagen deposition compared with vehicle-treated *Mdr2*-KO control mice (Figure 3d). The high abundance of α-SMA positive periductal cells found in *Mdr2*-KO mice [65] was also markedly reduced by CM414 treatment (Figure 3d). In response to the cholestatic injury, portal fibroblasts, which reside around the portal area and maintain the integrity of the biliary tree and portal tract, proliferate, get activated, and together with hepatic stellate cells, contribute to collagen type I deposition [66]. CD34, a marker of portal fibroblasts [62], was found highly expressed in control *Mdr2*-KO mice, predominantly in the portal and sinusoidal areas (Figure 3d). CD34 staining was markedly reduced in CM414-treated mice (Figure 3d). Ductular reaction, a cellular response associated with liver fibrosis and damage, and identified by CK19 staining, is commonly observed in *Mdr2*-KO mice [67]. We also observed a significant attenuation of this response in CM414-treated *Mdr2*-KO mice (Figure 3d).

**Figure 4:**
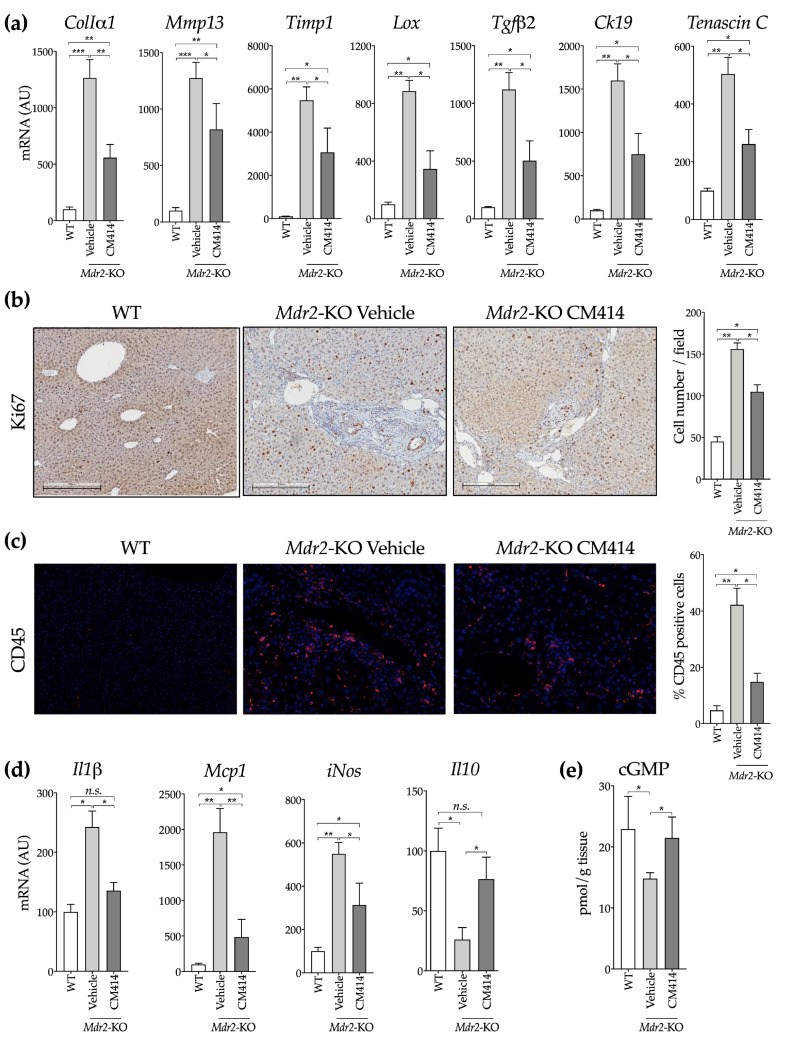
Evaluation of inflammation, fibrosis, and cell proliferation-related markers in the liver of WT, vehicle-treated *Mdr2*-KO, and CM414-treated *Mdr2*-KO mice. (**a**) qPCR analyses of the hepatic expression of fibrogenic activation-related genes. (**b**) representative immunohistochemical staining of Ki67 in liver sections. Graph on the right shows the quantification of Ki67 positive cells. (**c**) representative immunofluorescent staining of CD45 in liver sections. Graph on the right shows the quantification of CD45 positive cells. (**d**) qPCR analyses of the hepatic expression of inflammation-related genes. (**e**) analysis of cyclic guanosine monophosphate (cGMP) levels in mouse liver tissues. * *p* < 0.05. ** *p* < 0.01. *** *p* < 0.001.

Consistent with these histological observations, qPCR analyses of liver gene expression showed marked up-regulation of several genes associated with liver fibrogenesis, including collagen Iα1 (*ColIα1*), the interstitial collagenase matrix metalloproteinase 13 (*Mmp13*) and its inhibitor Timp1, lysyl oxidase (*Lox*) and transforming growth factor β2 (*Tgfβ2*), a TGFβ isoform strongly involved in biliary damage-related CLD [68] (Figure 4a). CM414 treated animals showed significantly reduced expression of all these genes, as that of *Krt19*, which encodes Ck19 and *Tnc*, which encodes tenascin C, a TGFβ-inducible extracellular protein expressed in liver fibrogenic cells that participates in inflammation, fibrogenesis, and carcinogenesis [69] (Figure 4a). Consistent with previous reports [65], we also observed enhanced hepatocellular proliferation in *Mdr2*-KO mice, as evidenced by increased hepatocyte and cholangiocyte staining with the proliferation marker Ki67, a response that was also reduced by CM414 treatment (Figure 4b).

Inflammation is a potent driver of liver fibrosis [14], and an inflammatory response is also observed in the liver of *Mdr2*-KO mice, contributing to liver injury and ECM deposition [63,70]. Therefore, we investigated the degree of hepatic inflammatory infiltration by staining for the leukocyte marker CD45. We found that CM414 treatment significantly reduced the amount of CD45 positive cells (Figure 4c), and this response was accompanied by a downregulation in the expression of proinflammatory cytokines, such as interleukin-1β (*Il-1β*) and monocyte chemoattractant protein 1 (*Mcp-1*), as well as the inducible NO synthase (*iNos*). Furthermore, the levels of the anti-inflammatory cytokine *Il10*, which are significantly reduced in *Mdr2*-KO compared with WT animals, were notably induced upon CM414 treatment (Figure 4d). Consistent with the inhibitory activity of CM414 towards PDE5, its administration to *Mdr2*-KO mice restored the hepatic contents of cGMP to levels similar to those found in WT animals (Figure 4e). All these results suggest that the simultaneous inhibition of Class I HDAC, HDAC6, and PDE5 activities results in a significant reduction in liver injury, fibrosis, and inflammation in *Mdr2*-KO mice.

### 2.2. Mechanisms of CM414 Anti-Fibrogenic and Anti-Inflammatory Effects

**Figure 5:**
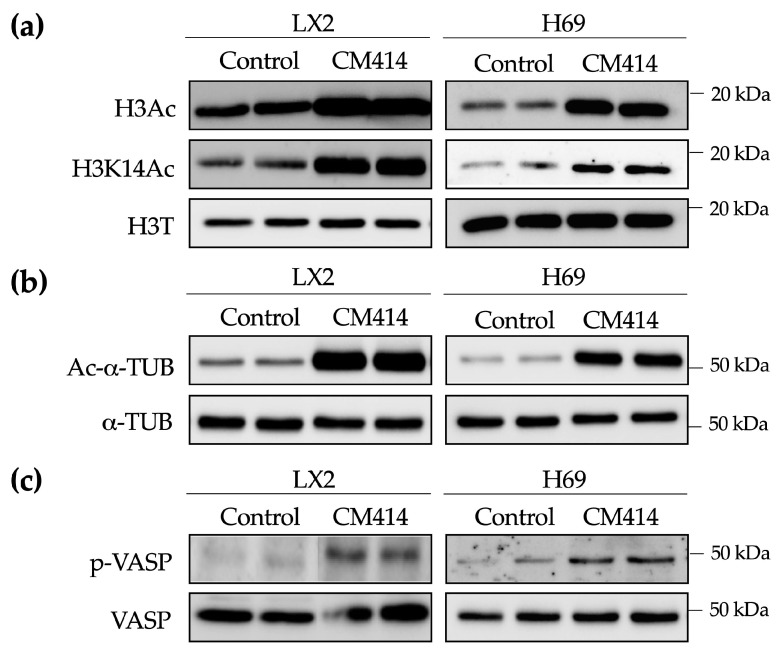
Evaluation of the on-target effects of CM414 in LX2 and H69 cells. (**a**) immunoblot analyses of total histone 3 acetylation (H3Ac), histone 3 acetylation at lys 14 (H3K14Ac) and total histone 3 (H3T) protein levels in LX2 (left panel) and H69 (right panel) cells treated for 1 h with CM414 (5 μM). (**b**) immunoblot analyses of acetylated-α-tubulin (Ac-α-TUB) and α-tubulin (α-TUB) protein levels in LX2 (left panel) and H69 (right panel) cells treated for 24 h with CM414 (5 μM). (**c**) immunoblot analyses of phospho-vasodilator-stimulated phosphoprotein (VASP) and VASP protein levels in LX2 (left panel) and H69 (right panel) cells treated for 24 h with CM414 (5 μM). Representative blots of three experiments performed in duplicate are shown.

To gain insight into the potential mechanisms of action of CM414 we used two different human cell lines, LX2 and H69, representative of liver ECM producing cells [71] and cholangiocytes [72], respectively. Similar to a previous report in neuroblastoma cells [56,57], in both LX2 and H69 cells, CM414 treatment resulted in a significant increase in total histone H3 lysine acetylation as well as histone H3 lysine 14 acetylation (H3K14Ac) levels (Figure 5a and Figure S1). Moreover, a significant increase was also observed in the levels of acetylated-α-tubulin, the best-characterized HDAC6 substrate [33], was also observed (Figure 5b and Figure S1). To test the PDE5 inhibitory capacity of CM414 we evaluated the phosphorylation levels of vasodilator-stimulated phosphoprotein (VASP). Upon PDE5 inhibition, intracellular cGMP levels increase, leading to the activation of PKG, a crucial downstream mediator of the cGMP-mediated signaling pathways [73,74]. VASP is a prime substrate of PKGs, and phosphorylation of VASP at Ser239 (p-VASP) is commonly used to asses PKG activity [75]. Consistently, LX2 and H69 cells treated with CM414 presented higher levels of p-VASP compared with control cells (Figure 5c and Figure S1). All these data support the on-target activity of CM414, in LX2 and H69 cells, i.e., inhibition of Class I HDAC, HDAC6, and PDE5.

**Figure 6:**
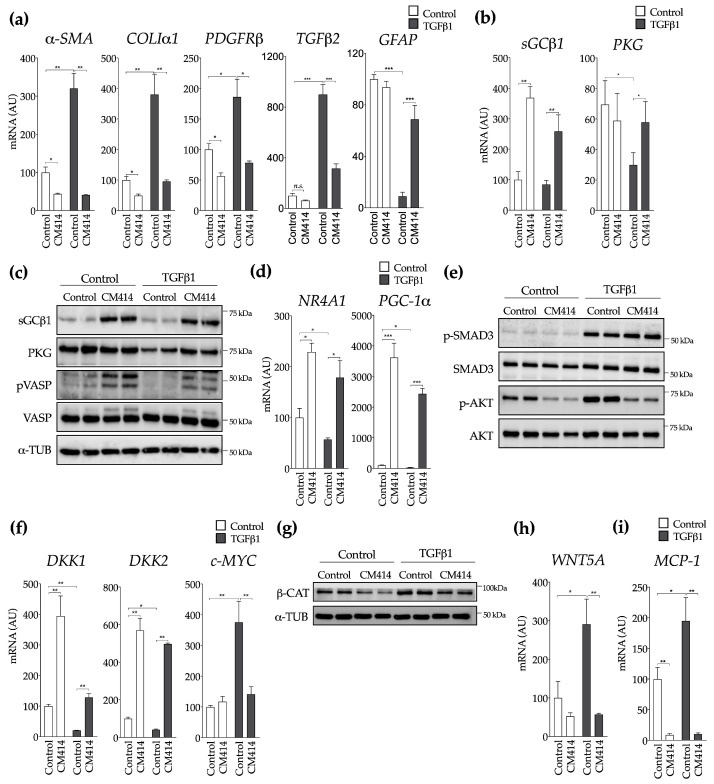
Effect of CM414 on transforming growth factor β (TGFβ)1-mediated activation of LX2 cells. In all experiments LX2 cells were treated with CM414 (5 μM) for 1 h and then stimulated with TGFβ1 (5 ng/mL) for another 24 h. (**a**) qPCR analysis of the expression of genes involved in fibrogenic activation. (**b**) qPCR analysis of the expression of sGCβ1 and protein kinase G (PKG). (**c**) immunoblot analysis of sGCβ1, PKG, phospho-VASP, VASP, and α-TUB protein levels in LX2 cells treated as indicated. (**d**) qPCR analysis of the expression of nuclear receptor 4 A1 (NR4A1) and proliferator activated receptor gamma co-activator-1α (PGC-1α) in LX2 cells treated as indicated. (**e**) immunoblot analyses of phospho-SMAD3, SMAD3, phospho-AKT and AKT protein levels in LX2 cells treated as indicated. (**f**) qPCR analyses of DKK1, DKK2, and c-MYC expression in LX2 cells treated as indicated. (**g**) immunoblot analyses of activated β-catenin (β-CAT) and α-TUB protein levels in LX2 cells treated as indicated. (**h**) qPCR analyses of *WNT-5A* expression in LX2 cells treated as indicated. (**i**) qPCR analysis of monocyte chemoattractant protein 1 (MCP1) mRNA expression in LX2 cells treated as indicated. Representative blots of three experiments performed in duplicate are shown. * *p* < 0.05. ** *p* < 0.01. *** *p* < 0.001.

To further elucidate the anti-fibrogenic mechanisms of CM414, LX2 cells were incubated with the key fibrogenic cytokine TGFβ1 in the presence or the absence of this compound. Consistent with our in vivo findings, we observed a marked impairment of TGFβ1 effects on the expression of fibrogenic genes, including *α-SMA*, *COLIα1*, *PDGFRβ,* and *TGFβ2* (Figure 6a). On the contrary, the inhibitory effects of TGFβ1 on the expression of glial fibrillary acidic protein (*GFAP*), a marker or quiescent ECM producing cells, were blunted by CM414 (Figure 6a). Interestingly, when we analyzed in detail different components of the sGC-cGMP-PKG axis, we found that the expression of sGC was potently upregulated even in the absence of TGFβ1 stimulation (Figure 6b,c and Figure S1). This finding suggests that in addition to preventing cGMP degradation through PDE5 inhibition, CM414 could also increase cGMP synthesis. The mRNA and protein levels of PKG were reduced upon TGFβ1 stimulation, strongly indicating that activation of myofibroblasts suppresses its expression, as previously described [76]. Concomitant treatment with CM414 and TGFβ1 resulted in the restoration of PKG levels (Figure 6b,c and Figure S1), and the levels of pVASP were also partially restored (Figure 6c and Figure S1). HDAC-mediated gene repression is also a critical event that precedes TGFβ1-induced pro-fibrotic gene expression. Transcriptional analyses revealed that TGFβ1 requires HDAC function to promote the repression of a broad complement of anti-fibrogenic genes [77]. We observed that specific anti-fibrogenic genes such as the orphan nuclear receptor 4 A1 (*NR4A1*) [78] or the metabolic regulator peroxisome proliferator activated receptor gamma co-activator-1α (*PGC-1α*), recently identified as a key guardian of fibroblasts quiescence [79], were potently reactivated upon CM414 treatment (Figure 6d). Interestingly CM414 did not seem to modulate the canonical TGFβ1 signaling pathway in LX2, as indicated by the unaltered phosphorylation of SMAD3 (Figure 6e). However, CM414 treatment resulted in a significant decrease in phosphorylated AKT (pAKT) levels, both in control cells and also after TGFβ1 stimulation (Figure 6e). The inhibition of this signaling pathway is consistent with the mechanism of action of other cGMP modulators endowed with anti-fibrogenic properties [53,54,80]. Nevertheless, to better understand the mechanisms underlying the inhibitory effect of CM414 on TGFβ1-AKT signaling we examined the expression of phosphatase and tensin homolog deleted on chromosome 10 (*PTEN*). PTEN is a negative regulator of the AKT pathway that is downregulated by epigenetic mechanisms in activated HSC, contributing to their activation [81]. We observed that CM414 treatment upregulated PTEN expression in LX2 cells (Figure S2a). Interestingly, CM414 also antagonized the upregulation of phosphatidylinositol-3 kinase (*PI3K*) catalytic subunit mediated by TGFβ1 (Figure S2b), a response that has been linked to the activation of lung fibroblasts and the development of pulmonary fibrosis [82,83].

Interestingly, we also found that CM414 significantly increased the expression of well-known endogenous antagonists of the WNT pathway [84], such as *DKK1* and *DKK2*, members of the Dikkopf family (Figure 6f). These observations may be of mechanistic relevance, as it has been recently established that WNT signaling cooperates with TGFβ-triggered pathways during fibrogenic activation of skin and lung fibroblasts [85]. Therefore, we first validated the crosstalk between TGFβ1 and WNT signaling in HSC activation. Here we show that when *DKK1* expression was knocked-down in LX2 cells the effects of TGFβ1 on the expression of genes relevant to their fibrogenic activation, such as *PDGFRβ*, *WNT-5A*, *c-MYC,* and *TGFβ1* itself, were markedly exacerbated (Figure S3). We confirmed that TGFβ1 reduced the expression levels of *DKK1* and *DKK2*, while CM414 treatment inhibited this response (Figure 6f and Figure S4). We evaluated the expression of *c-MYC*, a known target gene of WNT signaling, observing a significant increase upon TGFβ1 stimulation, and how this response was abated by CM414 (Figure 6f). Consistent with these observations, we found that the cellular levels of active β-catenin were downregulated upon CM414 treatment both in baseline conditions and after TGFβ1 stimulation (Figure 6g and Figure S1).Interestingly, we also observed that CM414 inhibited the induction of *WNT-5A* expression elicited by TGFβ1 treatment (Figure 6h). WNT-5A is a WNT family member that activates the non-canonical WNT pathways, and importantly it plays a relevant role in TGFβ1 mediated HSC activation and liver fibrosis [86,87]. Upon activation, hepatic stellate cells are also able to trigger an inflammatory response [16]. Therefore, we evaluated the expression levels of the critical cytokine monocyte chemoattractant protein 1 (MCP1). TGFβ1 stimulation increased *MCP1* expression in LX2 cells, and CM414 treatment also blunted this response (Figure 6i).

**Figure 7:**
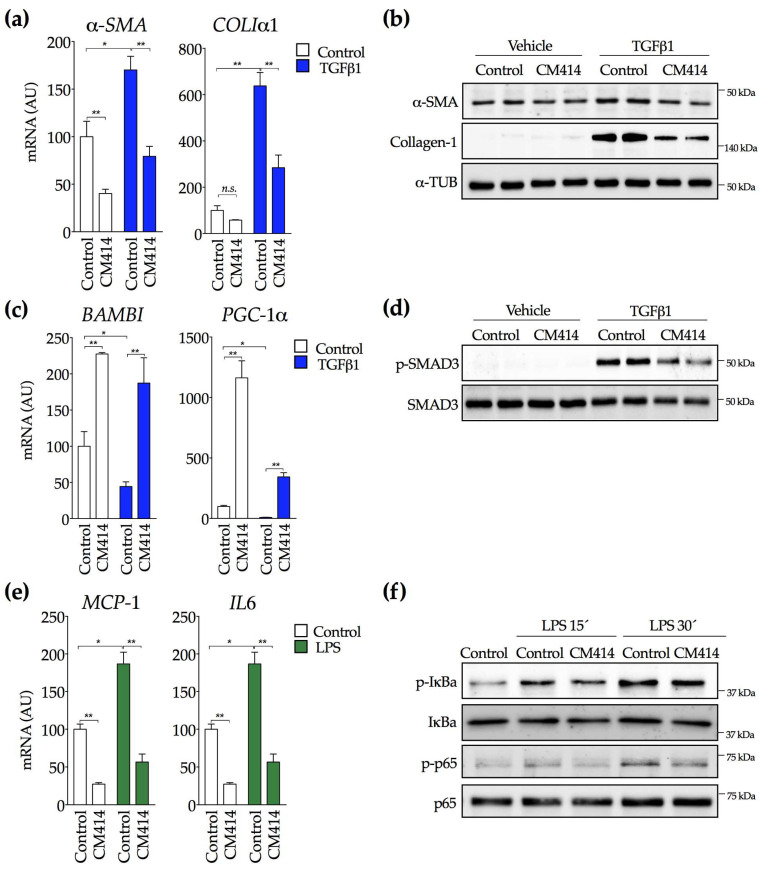
Effect of CM414 on TGFβ1 and lipopolysaccharide (LPS)-mediated activation of cholangiocytes. (**a**) qPCR analysis of *a-SMA* and collagen Iα1 (*COLIα*1) expression in H69 cells treated with CM414 (5 μM) for 24 h and then stimulated with TGFβ1 (5 ng/mL) for another 24 h. (**b**) immunoblot analyses of α-SMA, collagen-1 and α-tubulin protein levels in H69 cells treated as indicated above. (**c**) qPCR analyses of activin membrane-bound inhibitor (*BAMBI*) and *PGC-1α* expression in H69 cells treated as indicated above. (**d**) immunoblot analyses of phospho-SMAD3 and SMAD3 protein levels in H69 cells treated as indicated above. (**e**) qPCR analysis of *MCP1* and *IL6* mRNA expression in H69 cells treated with CM414 (5 μM) for 24 h and then stimulated with LPS (1 μg/mL) for another 24 h. (**f**) immunoblot analyses of phospho-IκBa, IκBa, phospho-p65 and p65 protein levels in H69 cells treated with CM414 (5 µM) for 24h and then stimulated with LPS (1 µg/mL for 15 min and 30 min. Representative blots of three experiments performed in duplicate are shown. * *p* < 0.05. ** *p* < 0.01.

In cholestatic liver injury and biliary fibrosis, diseased cholangiocytes become highly reactive, resulting in the release of several paracrine signaling molecules that subsequently activate portal fibroblasts and hepatic stellate cells in an epithelial/mesenchymal crosstalk [88,89]. To explore the potential effects of CM414 in this specific context, we used the human H69 cholangiocyte cell line. As previously described [90], H69 cells treated with TGFβ1 showed increased expression of the mesenchymal marker α-SMA along with a marked increase in collagen production. These effects were, significantly, blocked by CM414 treatment (Figure 7a,b and Figure S1). Consistent observations were made in primary mouse cholangiocytes, in which CM414 also inhibited the expression of *α-Sma*, *ColIα1*, *Pdgfrβ*, *Tenascin C,* and *Tnfα* elicited by TGFβ1 (Figure S5). Similar to what occurred in LX2 cells, CM414 potently induced the expression of know antagonists of the TGFβ pathway, such as the functional inhibitor of the TGFβ1 receptor, activin membrane-bound inhibitor (*BAMBI*) and *PGC-1α* (Figure 7c). Moreover, the downregulation of these two genes in response to TGFβ1 treatment was blunted by CM414 (Figure 7c). Interestingly, in contrast to LX2 cells, in H69 cells, CM414 attenuated SMAD3 phosphorylation in response to TGFβ1 (Figure 7d and Figure S1).

To further evaluate the anti-inflammatory properties of CM414, H69 cells were exposed to bacterial lipopolysaccharide (LPS) in order to trigger an inflammatory response. LPS significantly increased *MCP1* and *IL6* mRNA levels, whereas CM414 treatment completely prevented these responses. It is worth noting that CM414 markedly inhibited the expression of these genes even in the absence of LPS (Figure 7e). The NF-κb signaling system is a crucial intracellular mediator of inflammatory responses elicited by different stimuli, including LPS [91]. Therefore, we determined the effects of CM414 on the activation of this pathway. H69 cells were stimulated with LPS in the absence and the presence of CM414, and we examined the levels of IkBa and p65 phosphorylation (p-IκBa and p-p65) at different time points. As shown in Figure 7f (and Figure S1), CM414 attenuated the stimulatory effects of LPS on these two essential components of the NF-κb pathway. Moreover, the inhibitory effects of CM414 were also observed in the mouse macrophage cell line RAW264.7. CM414 attenuated the activation of the NF-κb pathway (p65 phosphorylation) by LPS and consistently reduced *Tnfα*, *Il1β,* and *iNos* expression (Figure S6). Together, these observations demonstrate that CM414 not only possesses potent anti-fibrogenic properties but also presents a significant anti-inflammatory activity through inhibition of the NF-κb pathway.

## 3. Discussion

Chronic hepatic injury and inflammation, regardless of its etiology, results in an unresolved wound healing process, which paves the way for liver cancer development [92,93]. Pharmacological interference with this dysregulated wound healing response may restore liver parenchymal architecture and function and prevent tumor development [94]. Here we have identified CM414, a first-in-class multi-targeted small molecule inhibitor as a potential tool to explore new anti-fibrogenic therapeutic avenues. The complex and multifactorial nature of liver fibrogenesis suggests that the simultaneous interference with different targets with a single molecule would likely be more effective. Moreover, considering that a deteriorated liver function is a hallmark of CLD, multi-targeted, but still, selective inhibitors may be less toxic than drug combinations, as pharmacological interactions would be avoided.

To test the anti-fibrogenic effects of CM414, we took advantage of *Mdr2*-KO mice, which as mentioned above, is a clinically relevant model of spontaneous CLD, fibrosis, and HCC/CCA development. First, we examined the expression of CM414 molecular targets in *Mdr2*-KO mice once inflammation and fibrosis are well established in this model [62]. Compared to WT mice, our IHC analyses clearly showed increased levels of HDAC1, HDAC6, and PDE5 in different liver cell types, including hepatocytes, ECM producing cells, and cholangiocytes. Next, we tested the effects of sustained CM414 administration on various parameters related to CLD progression. CM414 was well tolerated, which is an important issue when testing drugs in animals with impaired liver function. Moreover, CM414 dosing led to a decrease in liver-to-body weight ratio and improved serum markers of hepatic injury. This was accompanied by reduced hepatic fibrosis, reduced abundance of fibrogenic cells, and an attenuated ductular reaction. Accordingly, expression levels of genes implicated in myofibroblast activation, ECM accumulation, and overall tissue remodeling activity were lower in CM414 treated *Mdr2*-KO mice. Moreover, the levels of *TGFβ2*, previously shown to be specifically expressed in epithelial cells of proliferating bile ducts and fibrotic liver, contributing to ductular reaction, biliary damage, and fibrosis [68], were also reduced. Interestingly CM414 also lowered the expression of *Tenascin C* in *Mdr2*-KO treated mice. This ECM glycoprotein is minimally detected in healthy tissue but is transiently expressed during tissue injury, playing a role in fibrogenesis and also in tumorigenesis [69]. Tenascin C levels are significantly high in patients with chronic hepatitis C, liver cirrhosis, and HCC [95,96]. Its attenuation by CM414 could therefore identify a chemopreventive activity of CM414 towards HCC development. In line with this assumption, hepatocellular proliferation, which has been associated with enhanced risk of HCC development in chronic liver injury [97], was also diminished by CM414 treatment. The persistent inflammation associated with the progression of liver disease in *Mdr2*-KO mice was also attenuated, as indicated by decreased numbers of infiltrating leukocytes (CD45 positive cells) and reduced expression of inflammation-related genes. Moreover, CM414 restored the expression of the anti-inflammatory cytokine *Il10*, which is reduced in the liver of *Mdr2*-KO mice compared with WT animals. Taken together, these observations suggest that the concomitant inhibition of HDAC Class I, HDAC6, and PDE5 present not only anti-fibrogenic effects but also significant anti-inflammatory properties. Interestingly, we also observed that CM414 administration to *Mdr2*-KO mice restored the intrahepatic levels of cGMP to values similar to those found in WT animals. Enhanced intrahepatic cGMP availability has been recently observed in cirrhotic bile-duct ligated rats treated with the PDE5 inhibitor tadalafil. Tadalafil treatment improved liver fibrosis and inflammation, and also decreased portal pressure [98], suggesting that CM414 could also be useful for the management of portal hypertension [99].

From a mechanistic point of view, it is worth noting that the three different enzymatic inhibitory activities harbored by CM414 have been separately described to interfere with TGFβ signaling at multiple levels. Indeed, other Class I HDACs [27], HDAC6 [39], or PDE5 [80] inhibitors have been reported to hamper the proliferation and decrease the differentiation of liver fibroblasts into ECM-producing cells. We observed a significant impairment of TGFβ1-induced effects on the expression of crucial fibrogenic genes in LX2 cells treated with CM414. This response was not apparently due to interference with TGFβ1-SMAD signaling, as p-SMAD3 levels remained unaltered in the presence of CM414. However, Akt activation, a “non-canonical” TGFβ signaling pathway, also involved in the survival, proliferation, and collagen production of HSCs [100,101,102], was markedly inhibited by CM414. Interestingly, interference with TGFβ-mediated activation of Akt has also been observed in response to other histone deacetylase inhibitors, such as largazole [103], or upon PDE5 inhibition [80] in experimental models of liver fibrosis. The observed upregulation of *PTEN*, and the inhibition of TGFβ1-triggered *PI3K* gene expression, may also underlie the mechanisms through which CM414 interferes with TGFβ1-Akt signaling and HSC fibrogenic activation [81,82,83].

Interestingly we detected a significant increase in the expression of sGC in LX2 cells treated with CM414, both in the absence and the presence of TGFβ1. This effect may be attributed to an epigenetic mechanism mediated by Class I HDACs. Indeed, it has been shown that Class I HDAC inhibitors increase the expression of *sGC*, and more specifically, that HDAC3 represses its expression [104]. The fact that CM414 is able to upregulate the enzyme accounting for the production of cGMP, together with its intrinsic PDE5 inhibitory activity, makes this molecule a potent activator of cGMP-mediated signaling. This facet of CM414 pharmacological activity may certainly underlie the anti-fibrogenic effects observed in this study. Indeed, sGC stimulators are being actively studied due to their promising anti-fibrogenic actions not only in liver fibrosis [53] but also in other related conditions, such kidney fibrosis [105] and systemic sclerosis [54]. In these studies, it was also demonstrated that PKG activity was essential for the antifibrotic effects of sGC stimulators [106]. The impairment of the sGC-cGMP-PKG pathway in fibrotic tissues is phenocopied by TGFβ1 stimulation of ECM producing cells. In our hands, TGFβ1 treatment of LX2 cells significantly decreased PKG expression, while CM414 treatment averted this response. Together, these findings indicate that CM414 can have a multifaceted positive effect on the sGC-cGMP-PKG pathway, counteracting the inhibitory influence of TGFβ1 and thus, opposing the fibrogenic activation of liver myofibroblasts. On the other hand, through the inhibition of Class I HDACs, CM414 can favor the transcription of potential inhibitors of myofibroblast activation and fibrosis. In this respect, we observed a marked increase in the expression of genes such as *PGC-1α*, *NR4A1,* and *BAMBI* in LX2 or H69 cells upon CM414 treatment. Several studies have shown how TGFβ1 signaling leads to the repression of all these genes through epigenetic HDAC-dependent mechanisms [77,78,107]. Interestingly, we also observed that other negative regulators of pathways concomitantly triggered by TGFβ, such as the WNT signaling pathway, were upregulated upon CM414 treatment. The WNT/β catenin pathway also plays a crucial role in the progression of fibrosis [108], engaging in a pro-fibrogenic crosstalk with TGFβ signaling [85]. CM414 treatment led to the upregulation of the WNT antagonists DKK1 and DKK2, which are capable of suppressing the nuclear translocation of β-catenin and the induction of its transcriptional targets [84]. Other epigenetic mechanisms have been reported to participate in the transcriptional regulation of WNT antagonists [109]. The results of the present study have shown that Class I HDACs can also play a role, and HDACs inhibition could constitute a novel approach to interfere with the TGFβ/WNT signaling network in the context of liver fibrogenesis. Moreover, we also found that CM414 can interfere with the crosstalk between TGFβ1 and non-canonical WNT signaling. We observed that CM414 abrogated the effects of TGFβ1 on the expression of *WNT-5A*, a prominent growth factor of the non-canonical WNT pathway involved in liver fibrosis and TGFβ1-mediated HSC activation [86,87].

The epithelial to mesenchymal transition (EMT) has been identified as another biological process contributing to the pathogenesis of liver fibrosis [110]. In experimental models, EMT can be counteracted by HDACis, such as Class I HDACi, promoting the restoration of adherent junctions between epithelial cells [111], and HDAC6 inhibitors, which increase acetylated α-tubulin levels, resulting in microtubule stabilization [40,112]. In the *Mdr2*-KO model, EMT is particularly relevant, reproducing pathological features of human CLD [113]. Following liver injury and inflammation, cholangiocytes are activated and undergo trans-differentiation from epithelial to mesenchymal phenotype [110,114]. As mentioned above, CM414 treatment significantly attenuated the ductular reaction and the development of biliary fibrosis in *Mdr2*-KO mice. These responses may be due, in part, to a direct effect of CM414 on biliary epithelial cells. This contention is supported by our observations in H69 cells and primary mouse cholangiocytes. In these cells, TGFβ1 stimulation resulted in potent activation of a mesenchymal phenotype, including *α-SMA* expression and COLIα1 protein production, and interestingly, CM414 blocked this EMT-related response.

Finally, our in vivo and in vitro observations revealed remarkable anti-inflammatory properties for CM414, including the attenuation of parenchymal infiltration of inflammatory cells. Mechanistically, this effect could also be attributable to the different pharmacological activities of CM414. Several studies have reported how Class I HDAC [115], HDAC6 [36,37], and PDE5 [45,46] inhibitors present anti-inflammatory properties, which can be mediated by modulation of the NF-κB signaling pathway, as we observed in H69 cells and RAW264.7 macrophages. These anti-inflammatory activities may also contribute to the hepatoprotective effects of CM414 observed in *Mdr2*-KO mice, as inflammation is a key driver of liver damage in this model [63].

## 4. Materials and Methods

### 4.1. CM414

This molecule was designed using knowledge and structure-based approaches according to reported structure-activity relationships (SARs), available data regarding HDAC and PDE5 inhibition, and structural information, including the X-ray co-crystal structures for the HDAC2-vorinostat complex (PBD 4LXZ) and the PDE5-sildenafil complex (PDB 1TBF) (Chemical Computing Group, Molecular Operating Environment, MOE 2012.10 (2012), Montreal, Quebec, Canada; http://www.chemcomp.com/) [56]. Previous extensive multifactorial optimization of CM414 through medical chemistry, absorption, distribution, metabolism and excretion properties (ADME) studies, and cardiovascular safety and toxicity analyses highlighted this compound as an interesting pharmacological tool [58].

### 4.2. Animal Studies

Female *Mdr2*-KO− and *Mdr2*-WT mice (The Jackson Laboratory, BarHarbor, ME, USA) fed a standard laboratory diet were used in this study. Two littermates groups of 6-month-old *Mdr2*-KO mice were established. One group was treated daily with CM414 (40 mg/kg, i.p.) (*n* = 5) or vehicle (10% DMSO, 10% Tween-20 in saline solution) (*n* = 5) for 4 weeks. The control group of *Mdr2*-WT mice (*n* = 8) received the same volume of vehicle. This dose of CM414 was selected from a previous study [56]. At the end of treatments serum was withdrawn and animals were euthanized. Livers were excised, weighted, and were either span frozen of fixed in formalin and paraffin-embedded. Serums levels of liver enzymes levels were analyzed in a Cobas analyzer (Roche, Mannheim, Germany) as previously described [20,116,117,118]. cGMP levels in mouse liver tissues were measured with the Cyclic GMP ELISA kit from Cayman Chemical (581021) as recommended by the supplier. The study was approved by the Ethics Committee for Animal Experimentation of the University of Navarra (Protocol number 086-19).

### 4.3. Immunohistochemistry and Tissue Staining

Immunohistochemical detection of HDAC1 (antibody sc-81598, Santa Cruz Biotech, CA, USA), HDAC2 (sc-9959, Santa Cruz Biotech), HDAC6 (SAB500012, Sigma Aldrich, St. Louis, MO, USA), PDE5 (ab14672, Abcam, Cambridge, UK), α-SMA (A2547, Sigma Aldrich), CK-19 (ab9221, Abcam), Ki67 (ab15580, Abcam), CD45 (clone 30-F11,103101, BioLegend, San Diego, CA, USA), and CD34 (ab81289, Abcam) was performed on 3 μm thick formalin-fixed paraffin embedded mouse tissue following standard protocols as we described before [20,119]. Paraffin was removed and the tissues rehydrated using a slide wash/incubation sequence with Histo-Clear II (National Diagnostics, Nottingham, UK), ethanol 10%, 90%, and 70%, and ddH_2_O. Antigen retrieval was performed with Tris-EDTA Buffer/Citrate Buffer (Dako, Glostrup, Denmark) and sections were incubated with primary antibodies diluted in blocking solution (1% BSA in PBS) overnight at 4 °C. After washing, sections were incubated with secondary antibodies diluted in 1% BSA in PBS for another 1 h at room temperature and then washed and visualized with 3,3′-diaminobenzidine tetrahydrochloride (DAB) (Dako) counterstained with hematoxylin. The primary antibody for HDAC1 detection was diluted 1:100, HDAC2 1:100, HDAC3 1:100, HDAC6 1:100, PDE5 1:50, α-SMA (1:100), CK-19 (1:200), Ki67 (1:500), and CD45 (1:100), and the secondary antibody was anti-rabbit Envision + System-HRP (Dako) for all the antibodies used except Goat anti-rat Alexa Fluor 555 (A21434, Invitrogen, Carlsbad, CA, USA) for CD45. For microscopy and image analyses tissues were viewed under a Zeiss LSM 800 confocal microscope (Zeiss, Oberkchen, Germany). Images were processed and analyzed using ImageJ software [120].

### 4.4. Cell Culture and Treatments

The human HSC line LX2 [71] obtained from Millipore-Merck (Darmstadt, Germany), was cultured in Dulbecco’s Modified Eagle Medium (DMEM) supplemented with 2% FBS and 100 U/mL penicillin-streptomycin. H69 cells, an SV40-transformed (i.e., immortalized) human cholangiocyte cell line [72], were grown in DMEM Nutrient Mixture F-12 (DMEM/F-12) + GlutaMax supplemented with 10% FBS and 100 U/mL penicillin-streptomycin. RAW264.7 mouse macrophages were from the American Type Culture Collection (ATCC) and were cultured in DMEM supplemented with 10% FBS and 100 U/mL penicillin-streptomycin. Mouse cholangiocytes were isolated from *Mdr2*-WT male mice as we previously described [121] and cultured in thick collagen in fully supplemented DMEM/F-12 medium [122].

All cell cultures were maintained under standard conditions in a humidified incubator under 5% CO_2_ in air at 37 °C. TGFβ1 and lipopolysaccharide stimulation of LX2 and H69 cells was performed at 5 ng/mL and 1 μg/mL, respectively, while RAW264.7 cells were treated with 100 or 200 ng/mL LPS, as indicated. Recombinant human TGFβ1 was from R&D Systems (Minneapolis, MN, USA) and *Salmonella typhimurium* LPS from Sigma-Aldrich (St. Louis, MO, USA). In vitro treatments were performed at indicated times and doses, and controls received the same concentrations of DMSO (always <0.1% of final volume). For the knockdown of *DKK1* expression in LX2 cells we used control siRNAs (siC) and DKK1-specific siRNAs (siDKK1) from Santa Cruz Biotechnology (sc-37082). Transfections were performed as described [20].

### 4.5. Immunoblotting

Cells and tissues were lysed in RIPA buffer. Histones were extracted as described below. Samples were subjected to western blot analysis as reported [20,117]. Primary antibodies were: α-tubulin (T6074, Sigma-Aldrich), SMAD3 (C67H9, Cell Signaling, Danvers, MA, USA), pSMAD3 (C25A9, Cell Signaling), α-SMA (F3777, Sigma-Aldrich), VASP (3112, Cell Signaling), pVASP (Ser239) (3114, Cell Signaling), GUCY1B3, sGC1β (ab15484) (ab154841, Abcam), PKG-1 (3248, Cell Signaling), histone-3 (05-928, Millipore), Acetyl-Histone H3 (Lys14) (06-911, Millipore), pAKT (Ser473) (9271, Cell Signaling), AKT (9272, Cell Signaling), active β-catenin (05-665, Millipore), collagen-I (ab138492, Abcam, USA), and DKK1 (AF1096, R&D Systems). For quantification of immunoblot signals we used the Image Lab Software from Bio-Rad Laboratories.

### 4.6. RNA Isolation and Quantitative Real-Time RT-PCR

RNA was extracted from liver tissues and cells using the automated Maxwell system from Promega (Madison, WI, USA) according to the manufacturer’s instructions. For retro-transcription RNA samples were exposed for 1min at 90 °C for denaturalization followed by 1 h at 37 °C using a mix containing: 50 mM Tris-HCl pH 8.3, 75 mM KCl and 3 mM MgCl_2_, 10 ng/μL of random primers, 0.5 mM of each deoxyribonucleic triphosphate (dNTP), 5 mM of dithiothreitol (DTT), 1.2 U/μL RNase inhibitors (RNase out) and 6 U/μL of M-MLV inverse transcriptase enzyme. All reagents from Invitrogen (Carlsbad, CA, USA), except dNTPs that were from Roche Diagnostics (Mannheim, Germany). Resulting complementary DNA (cDNA) were used to measure differences among gene expression levels. With the resulting cDNAs qPCR reactions were performed in a Bio-Rad CFX96 Real-Time System thermal cycler using iQ SYBR Green Supermix reagent from Bio-Rad (Hercules, CA, USA) following manufacturer’s instructions. Primers are listed in the Table A1. Relative quantification of mRNA was calculated with the −ΔΔCT method using the reference gene of constitutive expression H3F3A as we described [20].

### 4.7. Histone Extraction

Histones were isolated as previously described [123]. Briefly, cells were lysed in a buffer containing 10 mM Tris-HCl pH 7.4, 10 mM NaCl, and 3 mM MgCl_2_. After centrifugation at 2500 rpm for 10 min at 4 °C supernatants were removed, and pellets were lysed in the previous buffer but containing 0.5% NP40 on ice for 10 min with gentle stirring. Nuclei were pelleted by centrifugation at 2500 rpm for 10 min at 4 °C and resuspended in 5 mM MgCl_2_ and 0.8 M HCl. Nuclei were incubated in this buffer during 30 min at 4 °C to extract the histones. Samples were then centrifuged at 14,000 rpm for 10min at 4 °C to pellet debris and supernatants were transferred to a clean tube where TCA 50% was added to precipitate the histones. After washing the pellets with acetone they were air-dried and resuspended in 100 mM Tris-HCl pH 7.5, 1 mM EDTA and 1% SDS. The histone concentration in the extract was measured using the BCA assay (Pierce Technologies, Rockford, IL, USA) according to manufacturer’s specifications.

### 4.8. Statistical Analyses

Data are means ± SEM. Data was compared using the Student *t* test. A *p* value of <0.05 was considered significant. Data analyses were performed using GraphPad Prism software version 7.0 (GraphPad Software Inc., San Diego, CA, USA).

## 5. Conclusions

**Figure 8:**
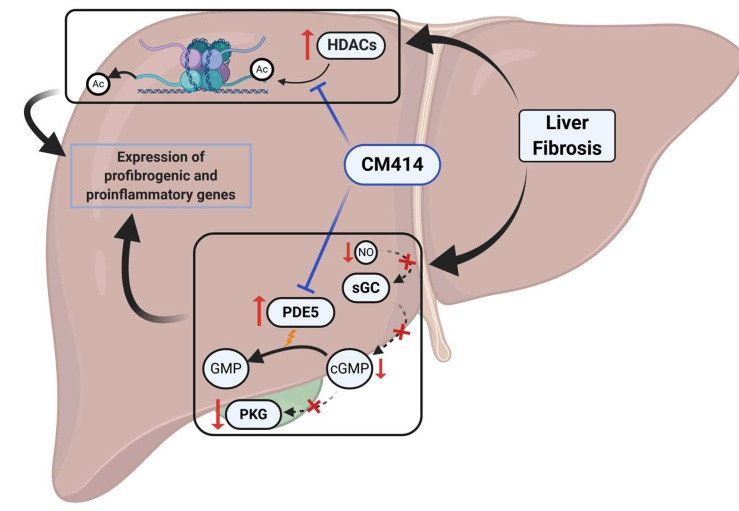
Proposed mechanisms of action of CM414 involved in the inhibition of liver fibrosis and inflammation.

Our study supports the notion that simultaneous targeting of different mechanisms involved in liver fibrosis and inflammation with a single molecule can be a new disease-modifying strategy. Here we illustrated this concept in a clinically relevant mouse model with established liver fibrosis using a novel class of multi-targeted molecular probes represented by CM414. Our findings show that combined inhibition of specific HDACs (Class I and HDAC6) and PDE5 results in potent anti-inflammatory and anti-fibrogenic effects in vivo in the absence of apparent toxicity (Figure 8). Mechanistically, our in vitro experiments indicate that inhibition of TGFβ expression and activity, restoration of the sGC-cGMP-PKG pathway, and attenuation of NF-κb inflammatory signaling may underly the beneficial effects of CM414. Therefore, the development of novel drugs based on compounds such as CM414 may open new avenues for the treatment of CLD and the prevention of malignant transformation of liver cells.

## Supplementary Materials

The following are available online at https://www.mdpi.com/2072-6694/12/12/3748/s1, Figure S1: Densitometric analyses of the immunoblots shown in Figure 5, Figure 6 and Figure 7, Figure S2: (a) effect of CM414 treatment on the expression of *PTEN* in LX2 cells treated for 48 h with CM414 (5 μM) analyzed by qPCR. (b) effect of TGFβ1 (5 ng/mL) on the expression of *PI3K* catalytic subunit in LX2 cells. Treatments were performed for 24 h in the absence or presence of CM414 (5 μM) as indicated, Figure S3: (a) qPCR analysis of *DKK1* gene expression in LX2 cells transfected with control siRNA (siC) or *DKK1*-specific siRNAs (siDKK1) 48 h after transfections. (b) qPCR analysis of the expression of *PDGFRβ*, *WNT-5A*, *c-MYC*, and *TGFβ1* in LX2 cells transfected with siGL or siDKK1 siRNAs (24 h) and then treated with TGFβ1 (5 ng/mL) for other 24 h as indicated, Figure S4: Immunoblot analysis of DKK1 expression in LX2 cells treated as indicated. CM414 (5 μM) was added 1 h before TGFβ1 (5 ng/mL) and cells were lysed for protein analyses 24 h later. α-tubulin (α-TUB) levels were analyzed as loading control. Representative blots are shown (left panel) and densitometric analyses of the immunoblots shown (right panel), Figure S5: qPCR analysis of the expression of *α-Sma, ColIα1, Pdgfrβ, Tenascin C,* and *Tnfα* in cultured primary mouse cholangiocytes treated with TGFβ1 (5 ng/mL) and CM414 (5 μM) for 24 h as indicated, Figure S6: (a) immunoblot analysis of phospho-p65 (p-p65) and p65 levels in RAW264.7 cells pretreated with CM414 (5 μM) for 2 h and then stimulated with LPS (200 ng/mL) for 15min. Representative blots are shown. (b) qPCR analysis of the expression of Tnfα in RAW264.7 cells pretreated with CM414 (5 μM) for 1 h and then stimulated with LPS (100 ng/mL) for the indicated times.

## Appendix A

**Table A1:** List of primers sequences.

Gene	Fw Sequence 5′-3′	Rev Sequence 5′-3′
*Ck19* (mouse)	CTCGGATTGAGGAGCTGAAC	TCACGCTCTGGATCTGTGAC
*ColIα1* (mouse)	CAGATTGAGAACATCCGCAG	GAATCCATCGGTCATGCTCTC
*H3f3a* (mouse)	AAAGCCGCTCGCAAGAGTGCG	ACTTGCCTCCTGCAAAGCAC
*Hdac1* (mouse)	CTACTACGACGGGGATGTTGG	GAATCTCTGCATCTGCTTGCT
*Hdac2* (mouse)	GAGGGATATTGGTGCTGGAA	AAGGGCAACTGCAGTCTCAT
*Hdac3* (mouse)	GAGAGTCAGCCCCACCAATA	GACCCGGTCAGTGAGGTAGA
*Hdac6* (mouse)	GCTGACTACATTGCTGCTTTCCT	ACTCCAGCATGGGGCAAG
*Il-1β* (mouse)	CATTGTGGCTGTGGAGAAGC	CCTTGTACAAAGCTCATGGAG
*Il-10* (mouse)	CTGCACCCACTTCCCAG	AGAAATCGATGACAGCG
*iNos* (mouse)	GTGAGGATCAAAAACTGG	TTTGCCTCTTTGAAGGAGC
*Lox* (mouse)	TAGGGCGGATGTCAGAGACT	CCAGGACTCAATCCCTGTGT
*Mcp1* (mouse)	CCACTCACCTGCTGCTACTC	TTCACATTCAAAGGTGCTGAAG
*Mmp13* (mouse)	CCTGATGTGGGTGAATACAATG	GGTTTCATCATCATCAAAATGG
*Pde5* (mouse)	GAAGGGGACCAGTGCTCAAG	CCACAATGCCTTTGTTCCA
*Tenascin C* (mouse)	GGATTGGTGTTTCTGCTGTCAA	TTTCAGACACCCGTAAGTCCTT
*Tgfβ2* (mouse)	CAGATCCTGAGCAAGCTGAA	CTGAAGTAGGGTCTGTAGAAAGTGG
*Timp1* (mouse)	AGACCACCTTATACCAGCG	AACAGGGAAACACTGTGCA
*Tnfα* (mouse)	GAGTGACAAGCCTGTAGCCC	CCCTTCTCCAGCTGGAAGAC
*Wnt-5a* (mouse)	GTCCTTTGAGATGGGTGGTATC	ACCTCTGGGTTAGGGAGTGTCT
*α-SMA* (human)	CCAGGGCTGTTTTCCCATCC	GTCATTTTCTCCCGGTTGGCC
*BAMBI* (human)	TGGATCGCCACTCCAGCTA	TGTCTTCATGACAGCATTCCA
*c-MYC* (human)	CTCAACGACAGCAGCTCGCC	CTTGAGGACCAGTGGGCTGTG
*COLIα1* (human)	CAGATTGAGAACATCCGCAG	GAATCCATCGGTCATGCTCTC
*DKK1* (human)	CAAAAATGTATCACACCAAAGG	TGGCTTGATGGTGATCTTTC
*DKK2* (human)	TGCCACAGTCCCCACCAA	TACAGACTTCCCCCTGATGG
*sGCβ1* (human)	GCAAGCATGCATCTGGAGAA	CCAGCAATTTCCATCATGTC
*GFAP* (human)	GAGATGATGGAGCTCAATGAC	TCCAGCCTCAGGTTGGTTTC
*H3F3A* (human)	AAAGCCGCTCGCAAGAGTGCG	ACTTGCCTCCTGCAAAGCAC
*IL-6* (human)	CCTTCCAAAGATGGCTGAAA	AAAGCTGCGCAGAATGAGAT
*MCP1* (human)	TCTGCCGCCCTTCTGTGCCTG	CTTCTTTGGGACACTTGCTGC
*PDGFRβ* (human)	GTGATGTGGGAACAGATGTCC	GAGGAAGCCCATGGTGGGATC
*PGC-1α* (human)	GCTGACAGATGGAGACGTGA	GTGTGAGGAGGGTCATCGTT
*PI3K* (human)	ATTGAACCAGTAGGCAACCG	GAAACTATTACCCAGATCACCAC
*PKG* (human)	CGCCAACCTGAAGCTGTCT	CCTTTGGAATGCAGATAGGC
*PTEN* (human)	GACTTAGACTTGACCTATATTTATCC	ACTCCCTTTTTGTCTCTGGTCC
*TGFβ2* (human)	CAGATCCTGAGCAAGCTGAA	CTGAAGTAGGGTCTGTAGAAAGTGG
*WNT-5A* (human)	GGGTGGGAACCAAGAAAAAT	TGGAACCTACCCATCCCATA
