# Supplementary material for: Dual Pharmacological Targeting of HDACs and PDE5 Inhibits Liver Disease Progression in a Mouse Model of Biliary Inflammation and Fibrosis
Source: Cancers (Basel). 2020 Dec 13;12(12):3748. doi: 10.3390/cancers12123748 (PMC7763137; doi:10.3390/cancers12123748)
Supplement: Supplementary file 1 [file cancers-12-03748-s001.zip › cancers-1041295-supplemental materials/cancers-1041295-Uncropped Western Blot Figures.pdf]

Figure 5a

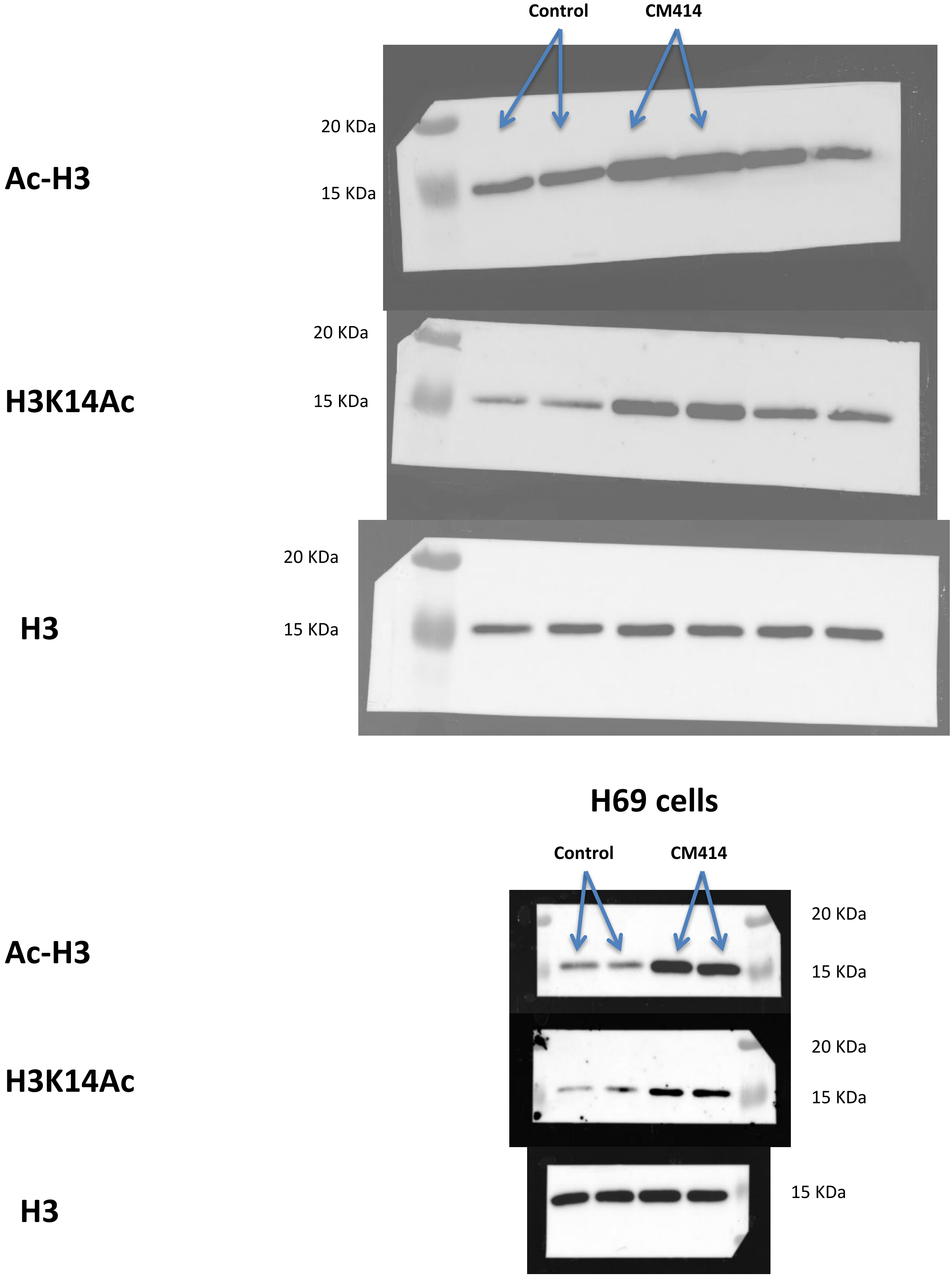
LX2 cells
Control
CM414
Ac-H3
20 KDa
15 KDa
H3K14Ac
20 KDa
15 KDa
H3
20 KDa
15 KDa
H69 cells
Control
CM414
Ac-H3
20 KDa
15 KDa
H3K14Ac
20 KDa
15 KDa
H3
15 KDa

Figure 5b

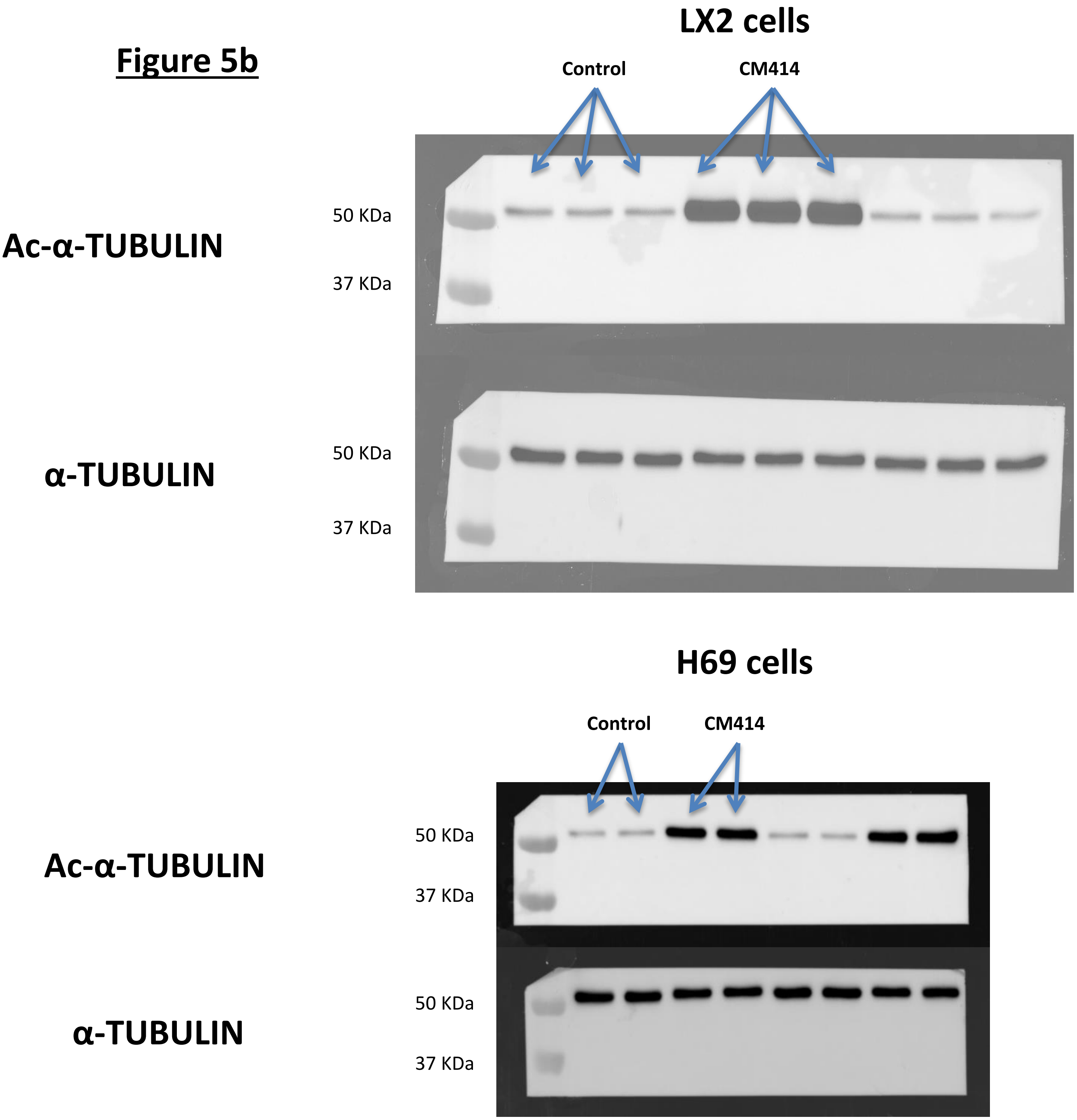
LX2 cells
Control
CM414
Ac-α-TUBULIN
50 KDa
37 KDa
α-TUBULIN
50 KDa
37 KDa
H69 cells
Control
CM414
Ac-α-TUBULIN
50 KDa
37 KDa
α-TUBULIN
50 KDa
37 KDa

Figure 5c

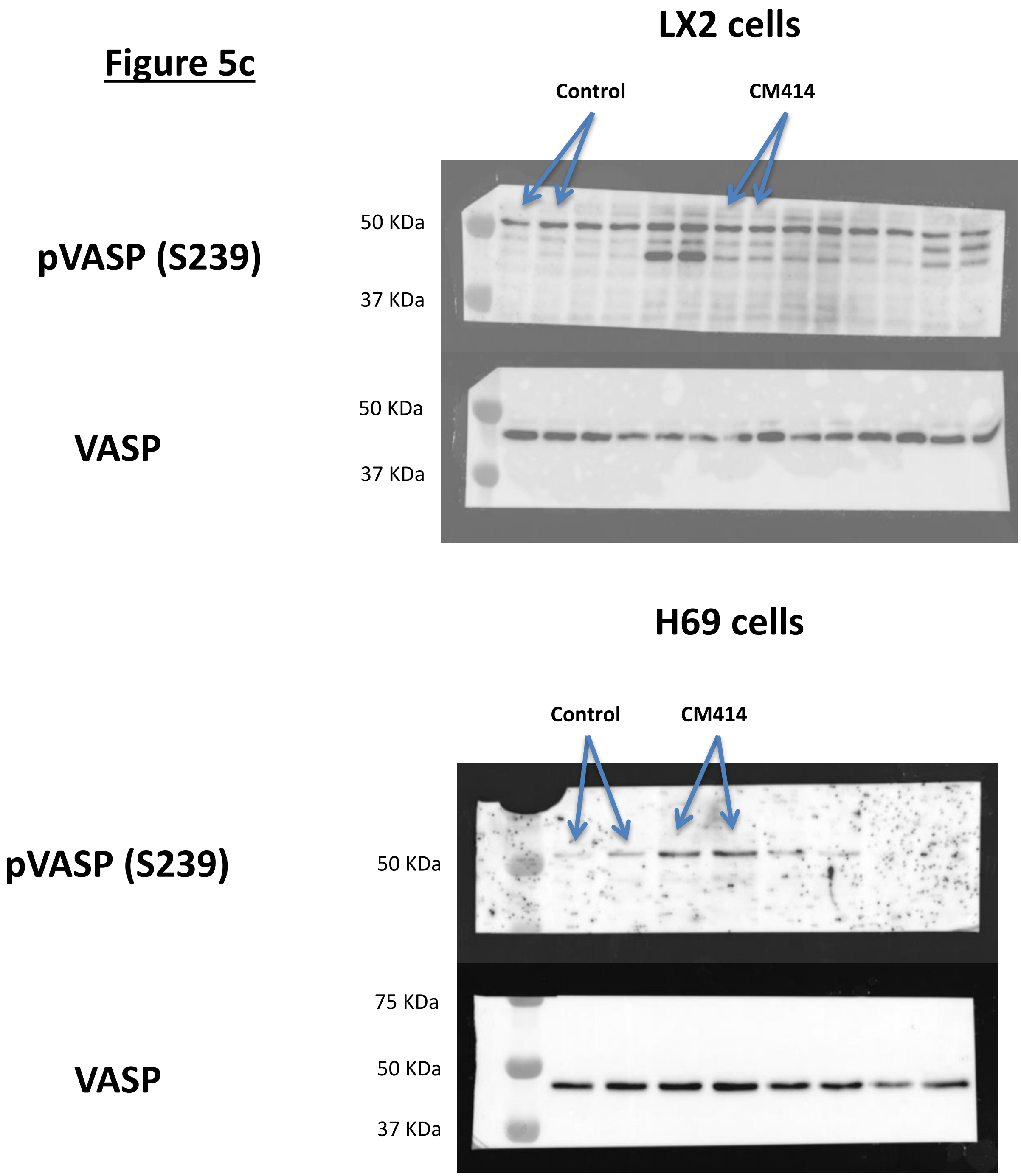

LX2 cells
Control
CM414
pVASP (S239)
50 KDa
37 KDa
VASP
50 KDa
37 KDa
H69 cells
Control
CM414
pVASP (S239)
50 KDa
VASP
75 KDa
50 KDa
37 KDa

Figure 6c

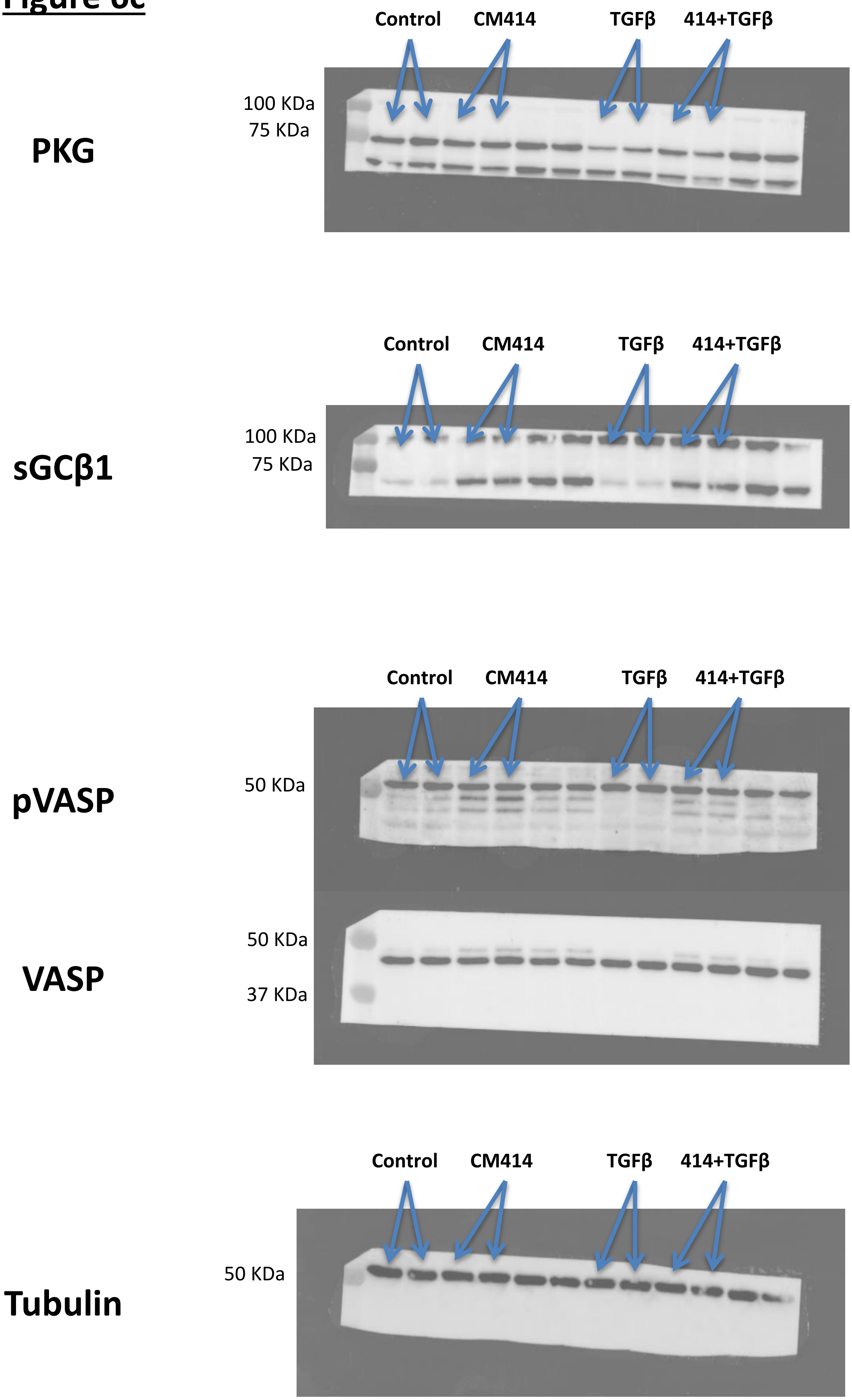

Control
CM414
TGFβ
414+TGFβ
PKG
100 KDa
75 KDa
sGCβ1
100 KDa
75 KDa
pVASP
50 KDa
VASP
50 KDa
37 KDa
Tubulin
50 KDa

Figure 6e

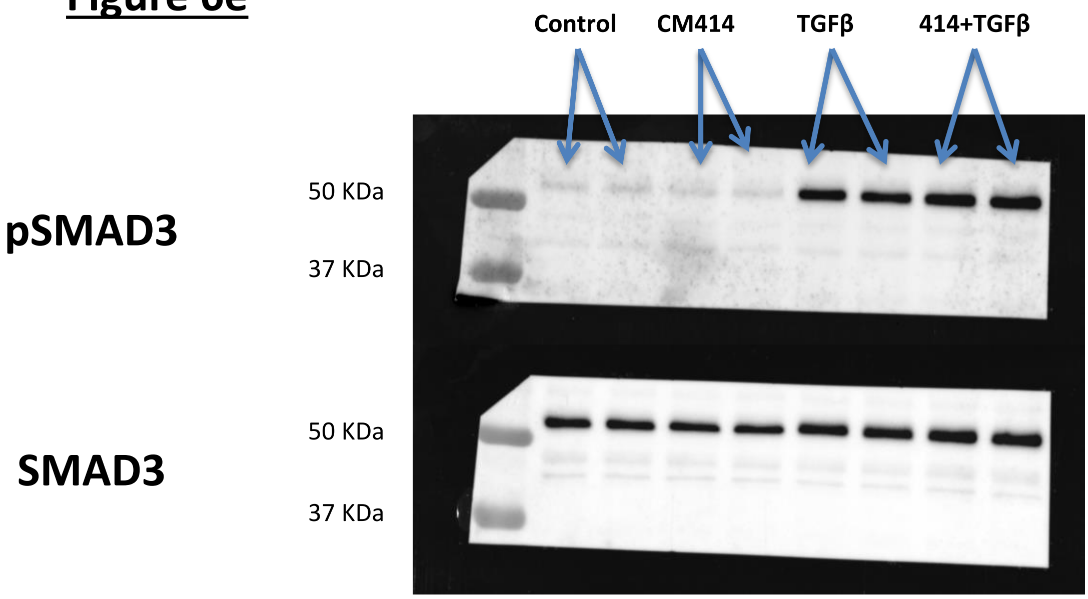

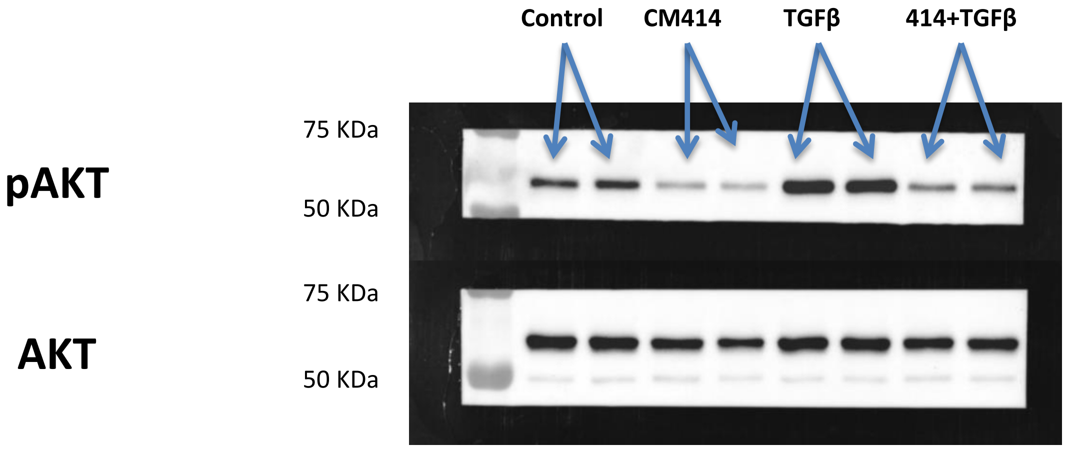

Figure 6g

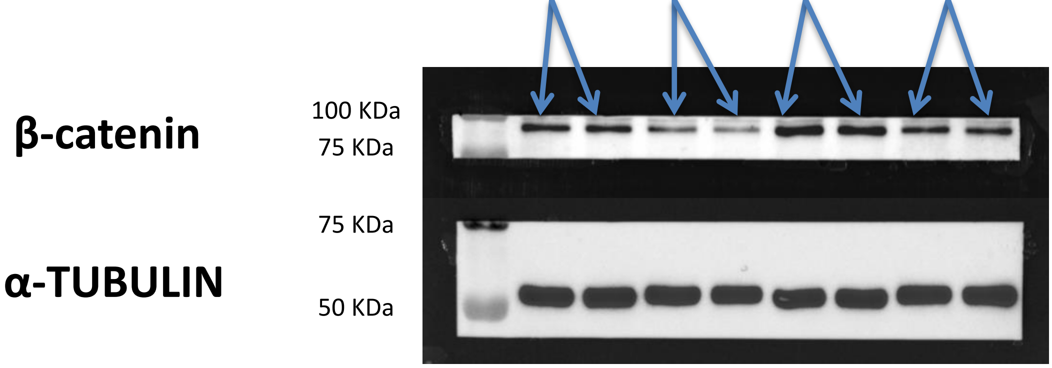

Figure 7b

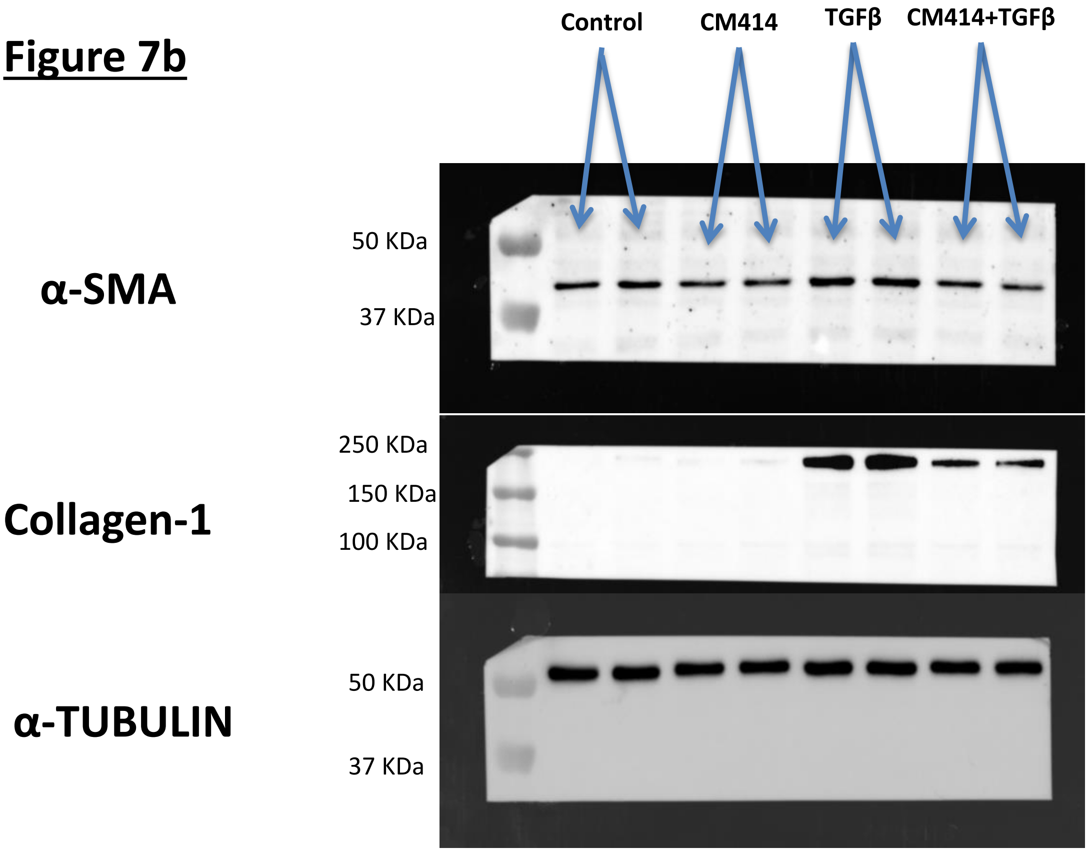

Figure 7d

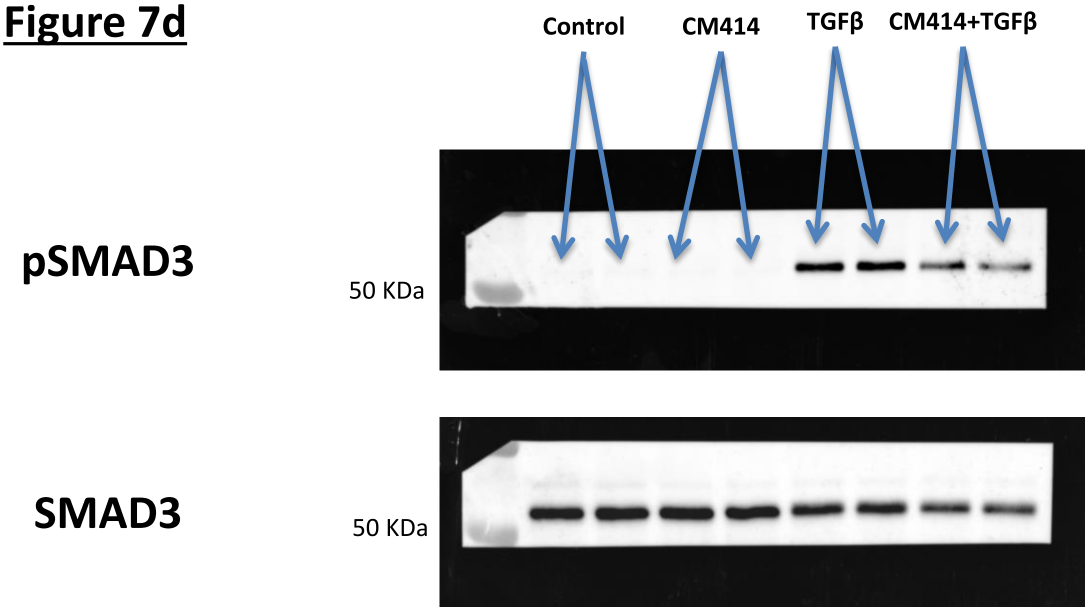

Figure 7f

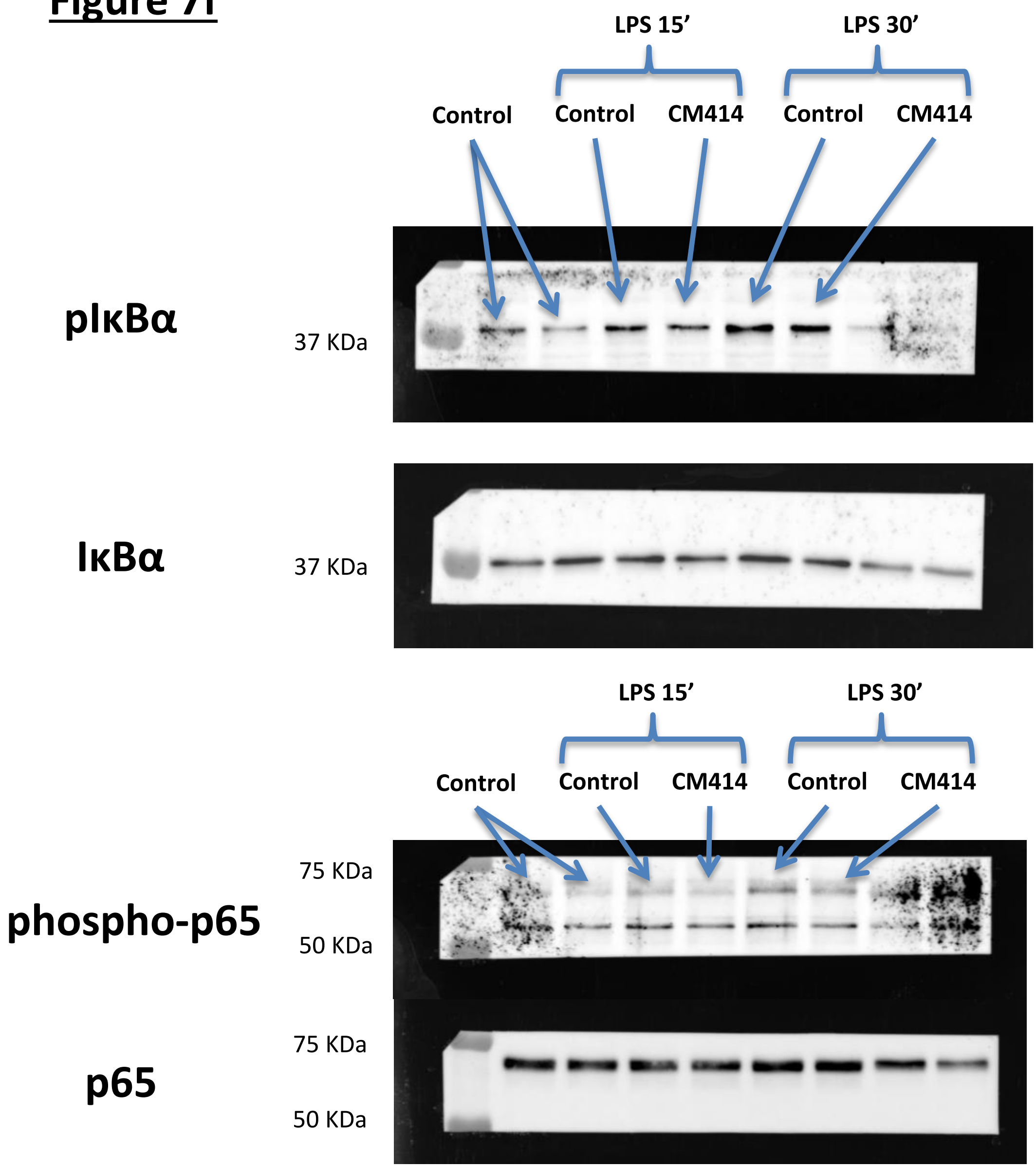
LPS 15'
LPS 30'
Control
Control
CM414
Control
CM414
pIκBα
37 KDa
IκBα
37 KDa
LPS 15'
LPS 30'
Control
Control
CM414
Control
CM414
phospho-p65
75 KDa
50 KDa
p65
75 KDa
50 KDa
