# Supplementary material for: Dual Pharmacological Targeting of HDACs and PDE5 Inhibits Liver Disease Progression in a Mouse Model of Biliary Inflammation and Fibrosis
Source: Cancers (Basel). 2020 Dec 13;12(12):3748. doi: 10.3390/cancers12123748 (PMC7763137; doi:10.3390/cancers12123748)
Supplement: Supplementary file 1 [file cancers-12-03748-s001.zip › cancers-1041295-supplemental materials/Supplementary Figures and Legends Claveria-Cabello et al-2.docx]

Supplemental Materials: Dual Pharmacological Targeting of HDACs and PDE5 Inhibits Liver Disease Progression in a Mouse Model of Biliary Inflammation and Fibrosis

Alex Claveria-Cabello, Leticia Colyn, Iker Uriarte, M. Ujue Latasa, Maria Arechederra, Jose M. Herranz, Laura Alvarez, Jesus M. Urman, Maria L. Martinez-Chantar, Jesus M. Banales, Bruno Sangro, Krista Rombouts, Julen Oyarzabal, Jose J. G. Marin, Carmen Berasain, Matias A. Avila and Maite G. Fernandez-Barrena


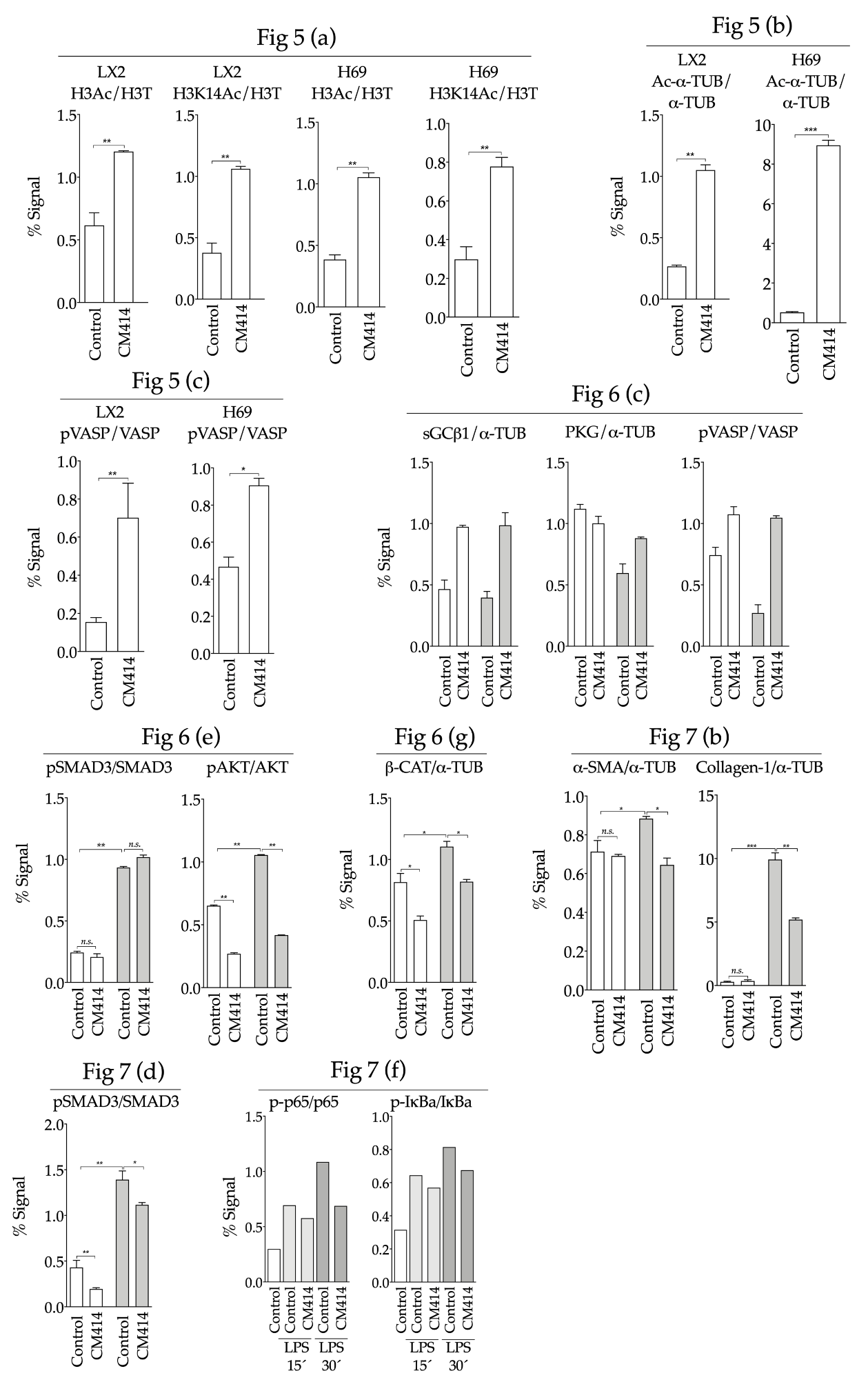


**Figure S1.** Densitometric analyses of the immunoblots shown in Figures 5, 6 and 7.


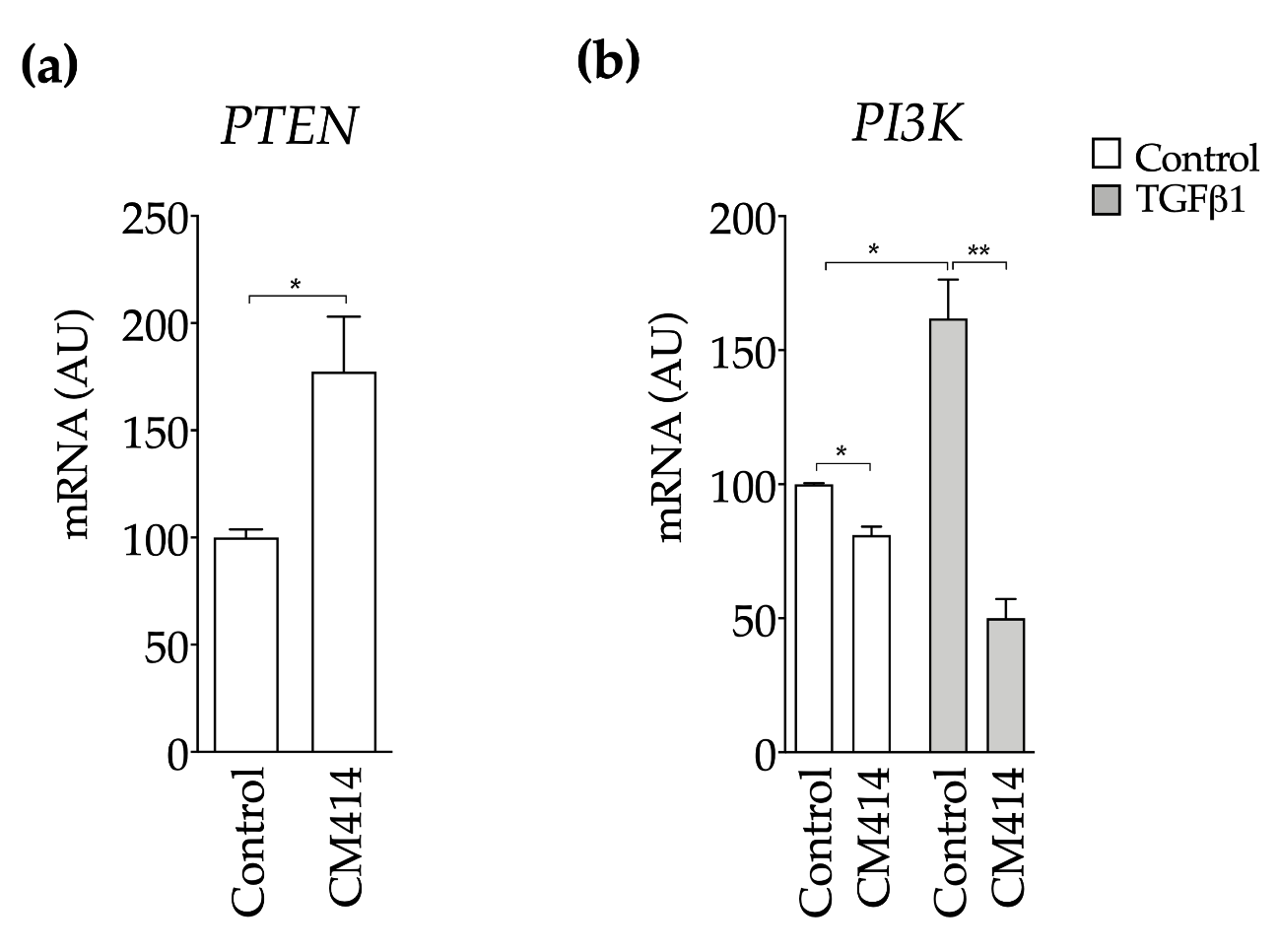


**Figure S2.** (**a**) Effect of CM414 treatment on the expression of *PTEN* in LX2 cells treated for 48 h with CM414 (5 μM) analyzed by qPCR. (**b**) Effect of TGFβ1 (5 ng/mL)on the expression of *PI3K* catalytic subunit in LX2 cells. Treatments were performed for 24 h in the absence or presence of CM414 (5 μM) as indicated. * *p* < 0.05, ** *p* < 0.01.


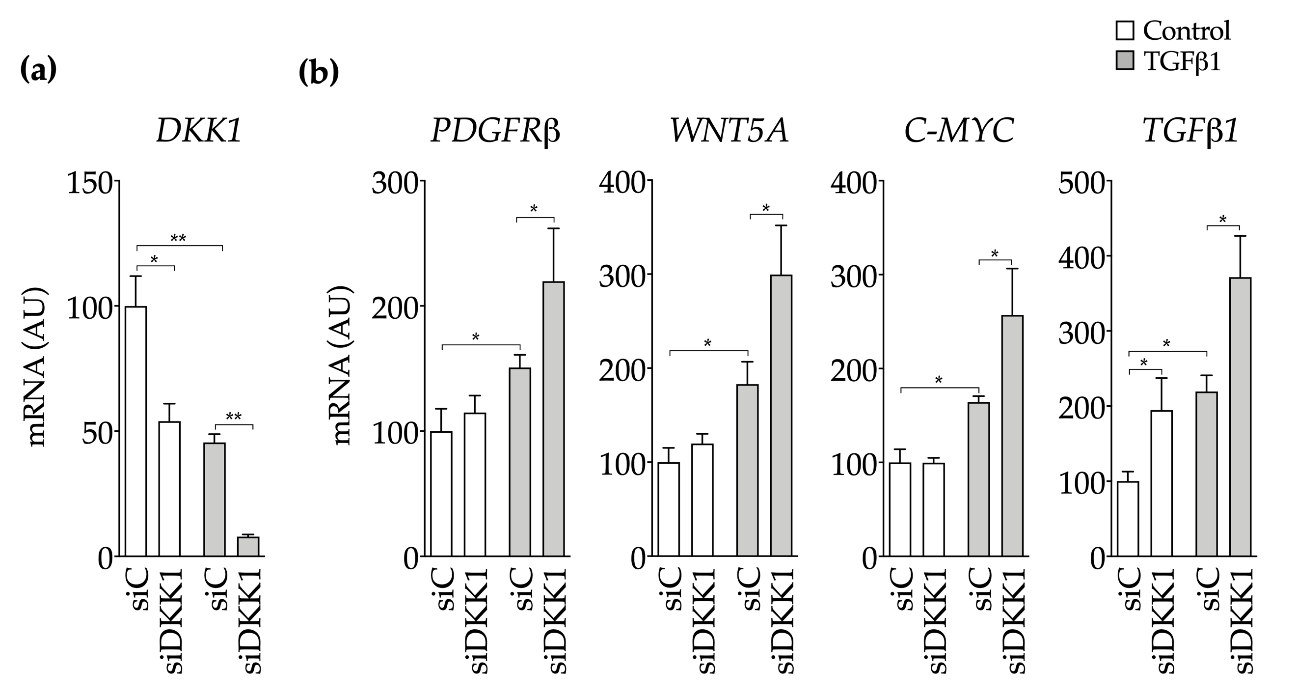


**Figure S3.** (**a**) qPCR analysis of *DKK1* gene expression in LX2 cells transfected with control siRNA (siC) or *DKK1*-specific siRNAs (siDKK1) 48 h after transfections. (**b**) qPCR analysis of the expression of *PDGFRβ*, *WNT-5A,* *c-MYC*, and *TGFβ1* in LX2 cells transfected with siGL or siDKK1 siRNAs (24 h) and then treated with TGFβ1 (5 ng/mL) for other 24 h as indicated. * *p* < 0.05, ** *p* < 0.01.


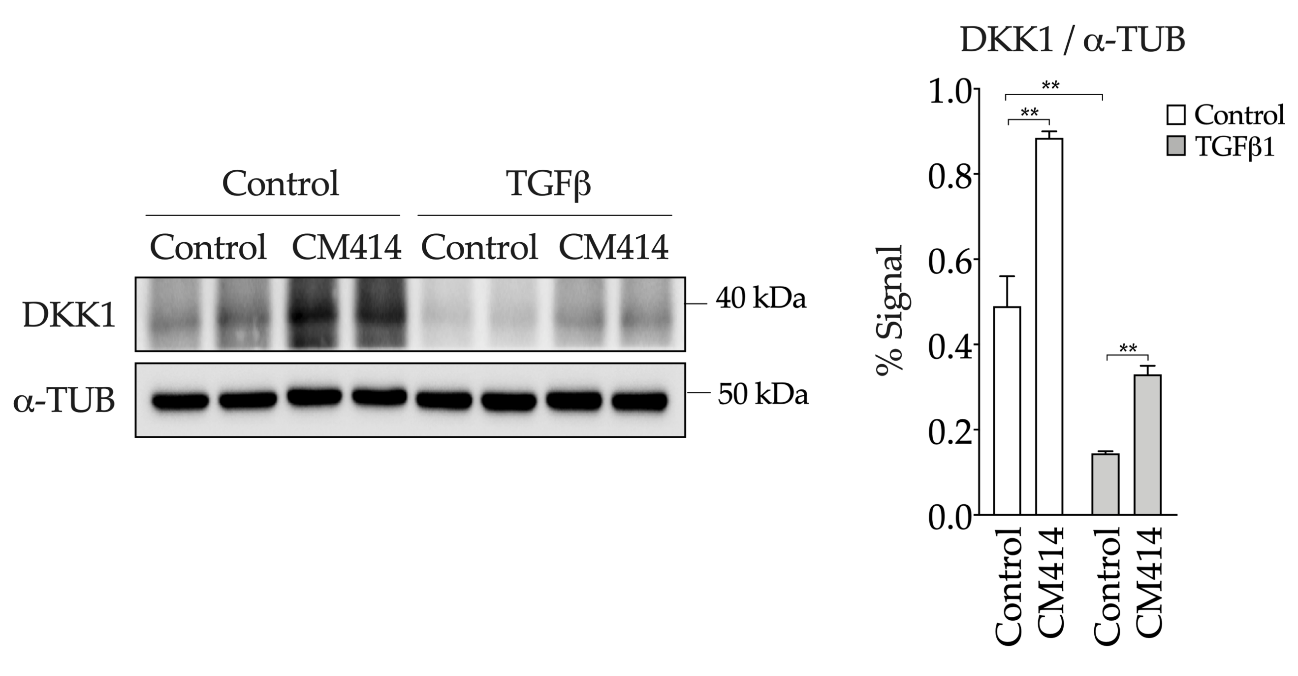


**Figure S4**. Immunoblot analysis of DKK1 expression in LX2 cells treated as indicated. CM414 (5 μM) was added 1h before TGFβ1 (5 ng/mL) and cells were lysed for protein analyses 24 h later. α-Tubulin (α-TUB)levels were analyzed as loading control.Representative blots are shown (left panel) and densitometric analyses of the immunoblots shown (right panel). ** *p* < 0.01.


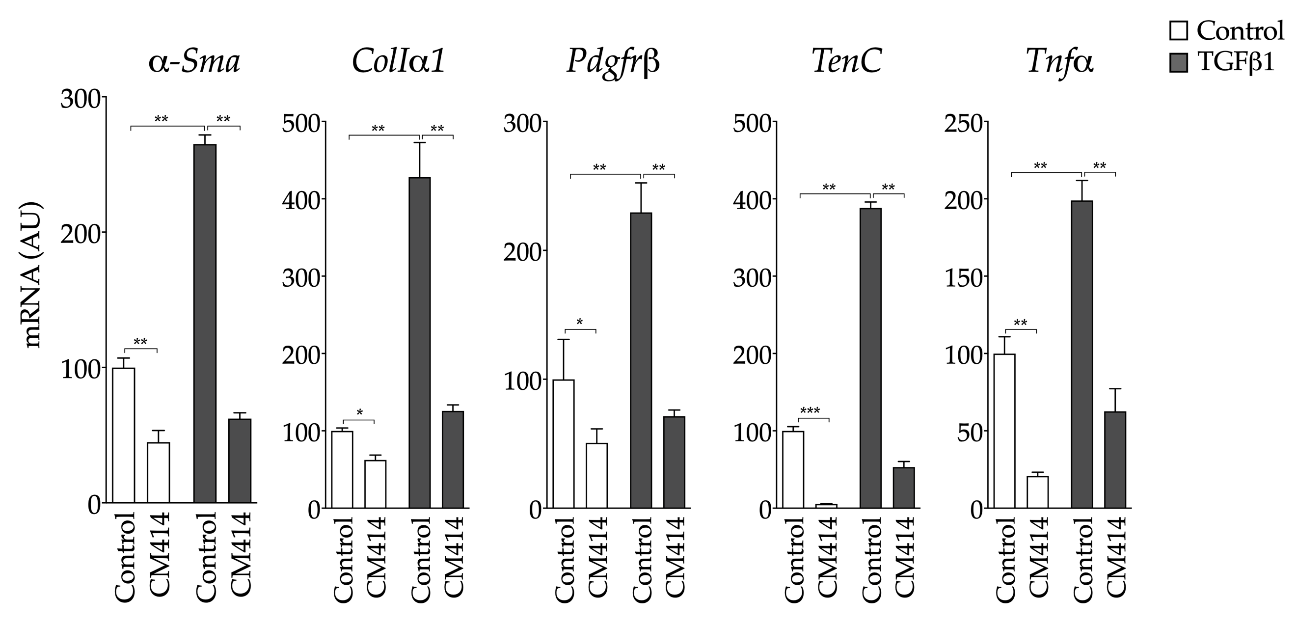


**Figure S5.** qPCR analysis of the expression of *α-Sma, ColIα1, Pdgfrβ, Tenascin C* and *Tnfα *in cultured primary mouse cholangiocytes treated with TGFβ1 (5 ng/mL) and CM414 (5 μM) for 24 h as indicated. * *p* < 0.05, ** *p* < 0.01, *** *p* < 0.001.

**Figure S6.** (**a**) Immunoblot analysis of phospho p65 (p-p65) and p65 levels in RAW264.7 cells pretreated with CM414 (5 μM) for 2 h and then stimulated with LPS (200 ng/mL) for 15 min. Representative blots are shown. (**b**) qPCR analysis of the expression of *Tnfα* in RAW264.7 cells pretreated with CM414 (5 μM) for 1 h and then stimulated with LPS (100 ng/mL) for the indicated times. * *p* < 0.05, ***p* < 0.01, *** *p* < 0.001.
